# Rapid biosensor development using plant hormone receptors as reprogrammable scaffolds

**DOI:** 10.1038/s41587-022-01364-5

**Published:** 2022-06-20

**Authors:** Jesús Beltrán, Paul J. Steiner, Matthew Bedewitz, Shuang Wei, Francis C. Peterson, Zongbo Li, Brigid E. Hughes, Zachary Hartley, Nicholas R. Robertson, Angélica V. Medina-Cucurella, Zachary T. Baumer, Alison C. Leonard, Sang-Youl Park, Brian F. Volkman, Dmitri A. Nusinow, Wenwan Zhong, Ian Wheeldon, Sean R. Cutler, Timothy A. Whitehead

**Affiliations:** 1 Department of Botany and Plant Sciences, University of California, Riverside, Riverside, CA USA; 2 Institute for Integrative Genome Biology, University of California, Riverside, Riverside, CA USA; 3 Department of Chemical and Biological Engineering, University of Colorado Boulder, Boulder, CO USA; 4 Department of Biochemistry, University of California, Riverside, Riverside, CA USA; 5 Department of Biochemistry, Medical College of Wisconsin, Milwaukee, WI USA; 6 Department of Chemistry, University of California, Riverside, Riverside, CA USA; 7 Department of Bioengineering, University of California, Riverside, Riverside, USA; 8 Department of Chemical Engineering and Materials Science, Michigan State University, East Lansing, MI USA; 9 Donald Danforth Plant Science Center, St. Louis, MO USA; 10 Department of Chemical and Environmental Engineering, University of California, Riverside, Riverside, CA USA; 11 Center for Plant Cell Biology, University of California, Riverside, Riverside, CA USA

**Keywords:** Molecular engineering, Plant hormones, Sensors and probes, Chemical tools

## Abstract

A general method to generate biosensors for user-defined molecules could provide detection tools for a wide range of biological applications. Here, we describe an approach for the rapid engineering of biosensors using PYR1 (Pyrabactin Resistance 1), a plant abscisic acid (ABA) receptor with a malleable ligand-binding pocket and a requirement for ligand-induced heterodimerization, which facilitates the construction of sense–response functions. We applied this platform to evolve 21 sensors with nanomolar to micromolar sensitivities for a range of small molecules, including structurally diverse natural and synthetic cannabinoids and several organophosphates. X-ray crystallography analysis revealed the mechanistic basis for new ligand recognition by an evolved cannabinoid receptor. We demonstrate that PYR1-derived receptors are readily ported to various ligand-responsive outputs, including enzyme-linked immunosorbent assay (ELISA)-like assays, luminescence by protein-fragment complementation and transcriptional circuits, all with picomolar to nanomolar sensitivity. PYR1 provides a scaffold for rapidly evolving new biosensors for diverse sense–response applications.

## Abstract (web summary)

Biosensors are developed from malleable hormone receptors.

## Main

Designing sensitive, specific and portable biosensors remains a difficult problem in biotechnology. A number of protein scaffolds have been co-opted from nature or designed from scratch to develop sensors for user-defined molecules, including bacterial allosteric transcription factors^1,2^, G-protein-coupled receptors^3^ and computationally redesigned binding proteins^4–6^, among others^7–9^. Each of these technologies has been successful in creating biosensors for a given application; however, they are limited by types of output signals available (e.g., transcriptional regulation) and rely on a limited palette of ligands. Methods to generate biosensors for user-defined molecules would accelerate many areas of biotechnology.

Chemically induced dimerization (CID) provides an appealing mechanism for coupling sensing to actuation; two proteins form a stable heterodimer only in the presence of a small molecule. Because they rely on a single protein–protein interaction, CID sensors can be used to build modular protein architectures that regulate transcription, control protein localization and degradation and modulate cell signaling^10^. However, in the CID systems typically exploited for sensing, ligand binding is shared between the binding partners, which necessitates redesign of the entire interface.

The ABA sensing system^11,12^ functions through a naturally occurring CID mechanism^10^ where ligand recognition by PYR1 leads to the formation of a stable PYR1–ligand–protein phosphatase (PP2C) complex that inhibits phosphatase activity. This system is unique, because ligand recognition occurs exclusively within PYR1, which simplifies the engineering of new CID modules. In addition, the phosphatase acts analogously to a coreceptor, because its binding to PYR1 lowers ligand off rates and boosts apparent affinity up to ~100-fold^13,14^. Thus, micromolar PYR1 binders in isolation can function as nanomolar sensors in combination with the phosphatase. Here, we exploit these beneficial traits using the *Arabidopsis* PYR1-PP2C system, PYR1-HAB1, to design sensors for diverse chemical classes. Our data show that high-density mutagenesis of this scaffold enables rapid development of sense–response actuators portable to in vitro and in vivo control systems.

## Results and discussion

**Fig. 1:**
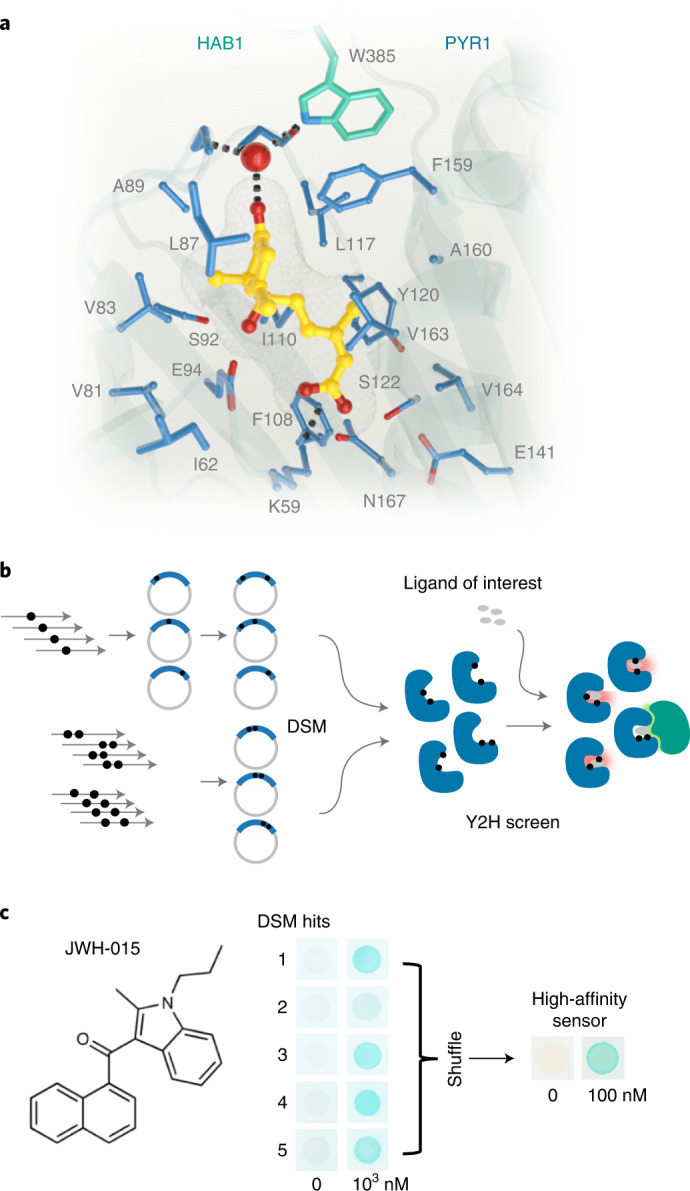
Protein structure-guided design of high-affinity PYR1-based cannabinoid sensors. **a**, The 19 side chains of residues in PYR1’s binding pocket targeted for double-site mutagenesis (DSM) are shown along with ABA (yellow) and HAB1’s W385 ‘lock’ residue and water network (3QN1). **b**, Sensor evolution pipeline. The PYR1 library was constructed by NM^12,15^ in two subpools, one using single-mutant oligos and another using double-mutant oligo pools. The combined pools were screened for sensors using Y2H growth selections in the presence of a ligand of interest. **c**, Representative screen results. The DSM library was screened for mutants that respond to the synthetic cannabinoid JWH-015 yielding five hits that were subsequently optimized by two rounds of DNA shuffling to yield PYR1^JWH-015^, which harbors four mutations. The yeast two-hybrid (Y2H) staining data show different receptor responses to JWH-015 by β-galactosidase activity.

We have previously shown that PYR1 can be repurposed to create an agrochemical receptor that functions in planta to modulate stress tolerance^11^; in that work, receptors were isolated from a library of variants created by single-site saturation mutagenesis. We reasoned that a structure-guided approach would facilitate the design of a larger collection of stable double mutants and ultimately enable the recognition of more ligands. There are 25 residues in PYR1’s ligand-binding pocket that make close contact to ABA and, therefore, 475 possible single mutants that can be combined to create 108,300 double mutants. We used our knowledge of the conserved receptor activation mechanism to remove six positions. To avoid combining unstable single mutants in the new library, we also used the Rosetta protein design software to predict the stability of the 361 single mutants at the remaining 19 positions. This analysis identified four positions that are particularly sensitive to mutation and were therefore restricted to a small number of amino acids in the final library; the remaining sites were allowed to mutate to any amino acid except cysteine or proline (Supporting File 1). Together, these steps condensed the library to a collection of 37,797 single and double nonsynonymous mutants (42,743 total mutants), which we constructed using site-directed mutagenesis. A total of 36,452 mutants were constructed using a pool of 301 single-mutant oligos in two sequential rounds of single-site nicking mutagenesis (NM)^15^; 6,291 of the mutants involved residues too close to one another (within eight residues) to be combined using single-mutant oligonucleotides and were instead constructed by NM using a pool of double-mutant oligonucleotides (Fig. 1b)^12^. The two libraries were combined to yield the double-site mutagenesis (DSM) pocket library for subsequent screening. The DSM library was deep sequenced and determined to possess >99.8% of the desired double mutants (Supporting File 1).

**Fig. 2:**
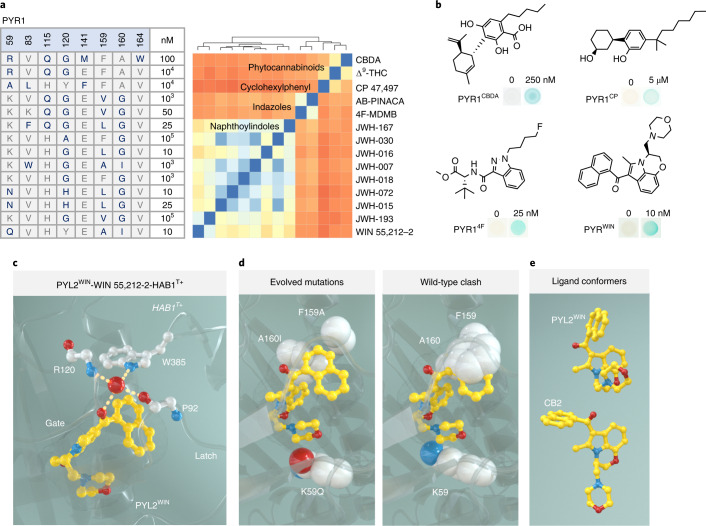
Sequence and structural basis of ligand recognition by evolved PYR1 sensors. **a**, Sequence diversity of cannabinoid receptor ligand-binding pocket residues (mutant residues are shown in bold type). The minimal ligand concentrations required for Y2H signal generation are indicated at right (Supplementary Fig. 2 shows full data, including mutations outside the pocket). The heatmap shows the ligands screened clustered by their pairwise Tanimoto distance scores calculated using ChemMine^33^; blue indicates high similarity, and orange has lower similarity. **b**, Representative optimized sensor Y2H β-galactosidase responses to the ligands indicated; PYR1^CBDA^ was evolved for recognition of CBDA, PYR1^CP^ for CP 47,497, PYR1^4F^ for 4F-MDMB and PYR1^WIN^ for WIN 55,212-2. **c**–**e**, Structural basis for cannabinoid recognition. **c**, WIN is colored yellow, and key ligand-contacting residues are indicated with dashes. The Trp-lock water network that stabilizes binding is shown at top. **d**, Relief of steric clash by the evolved receptor. **e**, Structural poses of WIN in PYL2-bound (top) and CB2-bound (bottom, 6PT0) structures.

**Extended Data Fig. 1:**
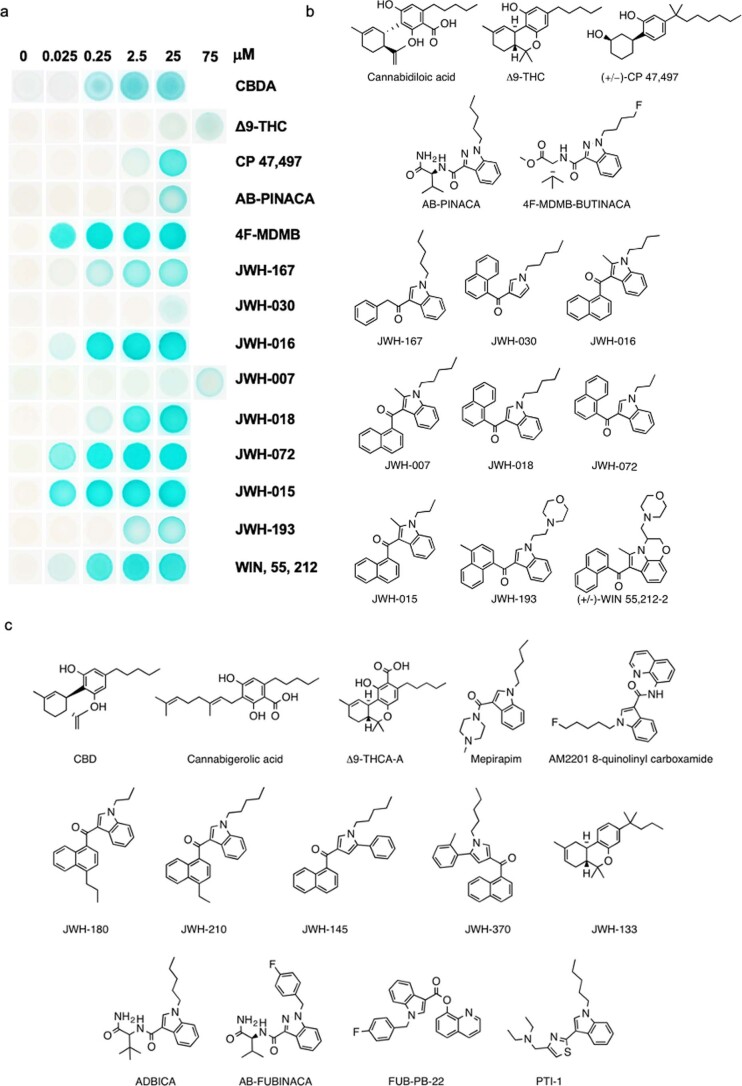
Chemical diversity of natural and synthetic cannabinoids sensed by engineered PYR1 sensors. (**a**) Y2H data of sensor hits; each row shows the response of an evolved PYR1 sensor to its target ligand; the sequences of the sensors used are shown in supporting data file 1. (**b**) Chemical structures of compounds for which at least one PYR1-based biosensor was identified. (**c**) Chemical structures of compounds for which no PYR1-based biosensor was identified.

With the improved library in hand, we set out to test its efficiency in a number of screens for biosensors. We first focused on developing cannabinoid sensors, in part to develop diagnostic reagents for synthetic cannabinoid mimics sold in products like ‘Spice,’ which have caused many hospitalizations^16^ and deaths^17,18^. We screened for PYR1 mutants responsive to any one of a panel of 28 cannabinoids, screening for mutants responsive to 30 μM of each test chemical (Extended Data Fig. 1 shows chemical structures). Selections were accomplished in a Y2H strain in which expression of URA3 rescues uracil auxotrophy via PYR1 binding to HAB1. Before selection, mutations that produced ligand-independent URA3 activation were removed by a negative selection in the presence of 5-fluoroorotic acid. Our initial positive selections identified double mutants responsive to JWH-015 (Fig. 1c) and five other naphthoylindoles, as well as cannabicyclohexanol (CP 47,497), a different chemical scaffold and one of the active ingredients in ‘Spice’; this demonstrates that our library can yield sensors in a single screening step. Additional sensors were acquired by iteratively screening diversified cannabinoid-biased sublibraries that were created by shuffling hit receptors against both the parental DSM and previous single-site mutant (SSM) libraries. Ultimately, these efforts identified 12 PYR1-derived cannabinoid receptors that recognize 14 compounds, including sensors for CBDA, Δ^9^-THC and 4F-MDMB-BUTINACA (4F-MDMB), from a total of 28 cannabinoids screened (Fig. 2a,b and Extended Data Fig. 1). Overall, mutations in nine out of the 19 residue positions targeted in the parental library were obtained in the cannabinoid receptors (K59, V81, V83, L87, A89, Y120, F159, A160 and V164), along with two additional sites (H115 and M158) present in the SSM library used for DNA shuffling and affinity maturation. We note that in two cases, our selections converged on the identical sequences; receptors responsive to JWH-072 and JWH-015, closely related naphthoylindoles that differ by only a single methyl substituent, yielded nearly identical evolved sequences. Similarly, the sensors obtained for the closely related compounds 4F-MDMB and AB-PINACA contained identical mutations (Supporting File 2). In addition, wild-type PYR1 did not respond to any of the target ligands screened, nor did our evolved high-affinity sensors respond to ABA (Supplementary Fig. 1); collectively, these data show that PYR1’s ligand-binding pocket can be reprogrammed to recognize target molecules.

Most synthetic cannabinoids share a central indole or indazole scaffold. We anticipated that our evolved cannabinoid receptors might show cross-reactivity. To explore this, we tested several high-affinity sensors for cross-reactivity to cognate ligands (Supplementary Figs. 1 and 2). Although these tests indicate some cross-reactivity, particularly for PYR1^4F^ and to a lesser degree PYR1^JHW-072^, in all cases, on-target sensitivity was at least tenfold higher than the off-target sensitivity. Thus, PYR1-derived sensors can provide sensitive and selective ligand detection, although this will vary by receptor and chemical.

To understand the underlying molecular basis for cannabinoid recognition by our evolved receptors, we sought to obtain the structure of a receptor–cannabinoid–HAB1 complex and targeted two high-affinity sensors, PYR1^4F^ and PYR1^WIN^. In our experience, PYL2 (a close relative of PYR1) forms crystals more readily than PYR1. PYL2 shares 88% pairwise sequence identity with PYR1 for the 25 ABA-proximal positions, and structures of the two proteins are globally alignable to 0.55 Å root mean squared deviation. We, therefore transposed mutations conferring ligand-selective responsiveness to PYL2, creating PYL2^4F^ and PYL2^WIN^, which both retain nanomolar ligand responsiveness (Supplementary Fig. 2). In addition, we created a stabilized, catalytically inactive derivative of HAB1 less prone to oxidative inactivation by employing computational redesign, yielding ΔN-HAB1^T+^ (derived from a HAB1 truncation that contains its PYR1-binding catalytic domain). This variant harbors 15 mutations, displays a ~7 °C increase in apparent T_m_, and retains high-affinity ligand-dependent binding to PYR1, as measured by a yeast surface display assay that uses PYR1^M^ (H60P, N90S), a monomeric double mutant optimized for yeast surface display^19^ (Supplementary Fig. 3 and Supporting File 1). Using these engineered proteins in matrix screens, we obtained diffraction quality crystals for a ternary PYL2^WIN^/WIN 55,212-2/ΔN-HAB1^T+^ complex, whose structure was solved by molecular replacement (1.9 Å resolution; Supplementary Table 1). Diffraction quality PYL2^4F^ crystals were not obtained. Several rounds of structural refinement were carried out before modeling WIN 55,212-2 into the ligand-binding pocket’s unbiased electron density (Supplementary Fig. 4). A real-space correlation coefficient of 0.967 calculated between the unbiased electron density and (+)-WIN 55,212-2 indicates agreement between the model and observed electron density. We note that the evolved receptor recognizes the biologically active (+)-WIN 55,212-2 stereoisomer, although selections were conducted using a racemate (Supplementary Fig. 2).

A central feature of ABA recognition by native sensors is the formation of a closed-receptor conformer that is stabilized by a hydrogen-bond network between a structurally conserved water, ABA’s ring ketone, main-chain amides in the gate and latch loops and HAB1’s W385 Trp-‘lock’ residue^20–22^. In our PYL2^WIN^ structure, WIN 55,212-2’s naphthoylindole ketone functions analogously to ABA’s ketone and is coordinated through water-mediated hydrogen bonds to backbone P92 in the gate, R120 in the latch and HAB1’s W385 lock residue (Fig. 2c).

Analysis of the PLY2-WIN structure also revealed that ligand binding is stabilized by an extensive network of hydrophobic contacts and a water-mediated contact to WIN’s morpholine oxygen (Supplementary Fig. 4). In comparison to PYR1, PYR1^WIN^ harbors three mutations (K59Q, F159A and A160I), and our structure illuminates their roles in allowing favorable binding. The most conspicuous effect is a relief of steric clash that would occur between F159 and WIN’s naphthalene ring in a wild-type receptor (Fig. 2d). The neighboring A160I mutation is positioned to enhance receptor-ligand surface complementarity by enabling the naphthoylindole to better pack in this position relative to the wild-type receptor. The K59Q mutation appears to reduce the electrostatic penalty of burying WIN’s positively charged morpholine ring but also organizes a water-mediated hydrogen-bond network at the base of the pocket (Fig. 2d and Supplementary Fig. 4). Thus, WIN’s binding to PYL2^WIN^ involves a combination of polar and hydrophobic contacts, which contrasts with its binding mode in the human cannabinoid receptor CB2, where binding involves exclusively hydrophobic contacts and a more extended ligand conformer^23^ (Fig. 2e). In addition, the structure illustrates the success of our HAB1 redesign, showing that ΔN-HAB1^T+^’s main chain is nearly superimposable with that of wild-type (0.85 Å root mean squared deviation) and that the key rotamers for residues involved in receptor interactions are maintained (Supplementary Fig. 3). Collectively, these data provide a mechanistic basis for the sensitive and selective cannabinoid recognition by our evolved receptor, illuminate the mutability of PYR1’s ligand-binding pocket and validate our computational HAB1 redesign.

**Fig. 3:**
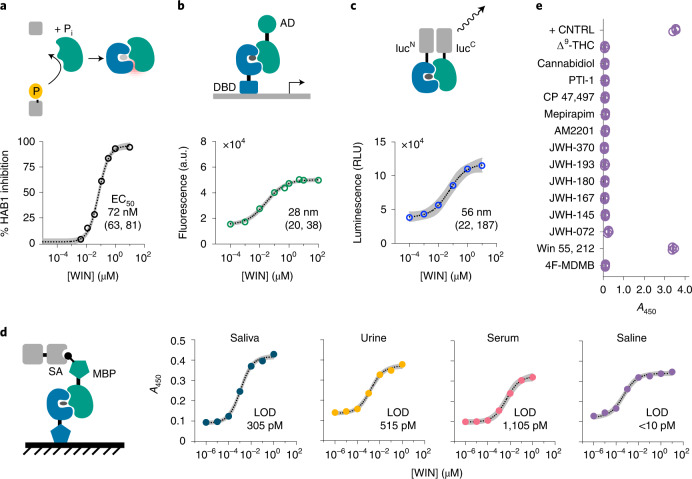
PYR1-based sensors are portable to diverse CID-based output systems demonstrated with PYR1^WIN^. **a**, Phosphatase inhibition. Ligand-dependent inhibition of ΔN-HAB1 phosphatase activity by recombinant receptors using a fluorogenic substrate. Inhibition expressed relative to mock controls (*n* = 3). **b**, Gene activation. Ligand-induced gene activation in *S. cerevisiae* using an engineered Z4-PYR1/VP64-ΔN-HAB1 genetic circuit. Whole-cell fluorescence generated from an integrated *Z4*_*4*_*-CYC1core-GFP-CYC1t* reporter is shown (12 h after ligand addition; *n* = 3). **c**, Split luciferase complementation. Addition of ligand results in luminescence from NLuc^N^-PYR1/NLuc^C^-ΔN-HAB1 (*n* = 3). **d**, PYR1 ELISA-like immunoassays. Immobilized receptors recruit biotinylated ΔN-HAB1^T+^ in response to ligand, and colorimetric signal is generated by a secondary streptavidin-HRP conjugate. Assays conducted in fivefold dilutions of saliva, urine, serum and blank saline are shown, with the lower limit of detection (LOD; Methods) of each assay shown (*n* = 3). Data points represent the mean, and the 95% confidence interval is shown on fits in **a**–**d** as gray shading and stated in square brackets along with the EC_50_ values. **e**, Receptor cross-reactivity evaluation in PYR1 ELISAs. The cannabinoids shown were assayed for signal generation at 2 µM. + CNTRL, PYR1^M^ tested with 2 µM ABA (*n* = 3); RLU, relative luminescence unit. Protein parts: DBD, DNA binding domain; AD, activation domain; MBP, maltose binding protein; SA, streptavidin. Chemicals: THC, tetrahydrocannabinol; WIN, (+)-WIN 55,212-2. Source data

In principle, the PYR1-HAB1 CID mechanism enables rapid construction of multiple sense-response outputs, as has been demonstrated with other designed CID sensors^1^. To explore the portability of the designed PYR1-HAB1 system, we selected two high-affinity receptors for evaluation of in vitro HAB1 inhibition, yeast transcriptional activation circuits and in vivo protein-fragment complementation with split luciferase. PYR1^WIN^ and PYR1^4F^ displayed nanomolar half-maximum effective concentration (EC_50_) values using an inhibition assay that detects receptor activation by changes in HAB1 phosphatase activity using a fluorogenic substrate (EC_50_ PYR1^WIN^: 72 nM; PYR1^4F^: 102 nM; Fig. 3 and Supplementary Fig. 5). Fusion of the transcriptional activator VP64 to ΔN-HAB1 and a zinc-finger DNA-binding domain to PYR1^WIN^ enabled inducible GFP expression from a synthetic cassette integrated into the genome of *Saccharomyces* *cerevisiae* with an EC_50_ of 28 nM. The same transcriptional circuit built with PYR1^4F^ responded to 4F-MDMB with an EC_50_ of 23 nM. Similar success was achieved with the NanoLuc split luciferase systems^24^; NLuc^N^-PYR1^WIN^/NLuc^C^-ΔN-HAB1 responded with an EC_50_ of 56 nM, and NLuc^N^-PYR1^4F^/NLuc^C^-ΔN-HAB1 responded with an EC_50_ of 25 nM. Taken together, these results show that the PYR1/ΔN-HAB1 CID mechanism enables portability to both in vitro and in vivo formats and can be deployed in multiple outputs. Moreover, the luminescence and fluorescence reporting modes may be advantageous for ligand detection using low-cost instrumentation and/or in remote settings using engineered living cells.

Synthetic cannabinoids are frequently modified to evade detection by routine drug testing. For example, 4F-MDMB is a relatively new indazole that first appeared in 2018 and rapidly became one of the most prevalent synthetic cannabinoids used in the United States^25–27^. Although mass spectrometry methods can sensitively detect this and most synthetic cannabinoids, lower-cost and easier-to-use immunoassays (e.g., ELISAs) dominate routine drug testing. Given this, we sought to convert our CID system into an ELISA-like system for microplate format measurements. To do so, we developed a hybrid sandwich-assay in which the PYR1 sensor is surface immobilized and then coincubated with biotinylated ΔN-HAB1^T+^ and the ligand of interest. Detection of ternary complexes can then be quantified using streptavidin-linked horseradish peroxidase (HRP), as is commonly done with ELISAs. We developed an ABA-detection system using PYR1^M^ (LOD 2 nM, 0.7 ng ml^−1^) and then adopted this optimized format for PYR1^4F-M^ and PYR1^WIN-M^ (Supplementary Fig. 5 and Supporting File 1). To evaluate the assay’s performance in forensically relevant samples, we tested it with spiked urine, blood and saliva samples and observed reliable detection of picomolar concentrations (LOD: 515 pM in urine, 1,105 pM in blood serum and 305 pM in saliva; Fig. 3d), which compares favorably with existing immunoassay kits that typically report low-nanomolar LODs^28^. In addition, minimal cross-reactivity between the PYR1^WIN-M^ and PYR1^4F-M^ sensors and a panel of 14 cannabinoids was observed using this detection format (Fig. 3e and Supplementary Fig. 5). Combined with our other results, these data further demonstrate that PYR1’s native CID mechanism can be harnessed to develop multiple sense-response outputs and demonstrate that our optimized ELISA-like test enables selective and sensitive detection of target ligands using evolved PYR1-based sensors.

**Fig. 4:**
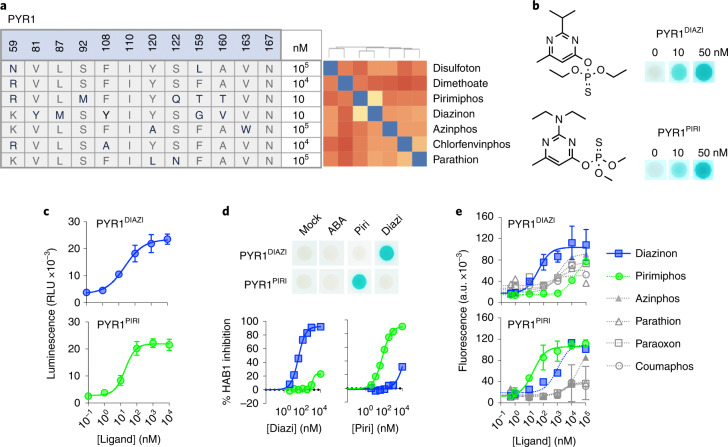
Facile development of potent, selective and portable organophosphate sensors. **a**, Summary of biosensor screening results for a panel of ten organophosphates. The compounds screened are clustered by similarity (blue indicates more similar) using a distance matrix of pairwise Tanimoto similarity scores, calculated in ChemMine 19. The molecules that yielded hits are shown in bold type; the minimal ligand concentrations required for Y2H signal generation for optimized receptors (Methods) are indicated (Supplementary Fig. 12 shows additional details). **b**, ﻿The optimized PYR1^DIAZI^ and PYR1^PIRI^ are high-affinity sensors. Optimized receptors were tested for responses to nanomolar concentrations of diazinon and pirimiphos-methyl, respectively, as evidenced by Y2H assays and receptor-mediated inhibition of HAB1 phosphatase activity in vitro. PYR1^DIAZI^ (EC_50_ = 36 nM [32,40]); PYR1^PIRI^ (EC_50_ = 58 nM [50,67]). Wild-type PYR1 was used as a control (gray lines). **c**, PYR1-derived receptors are portable. PYR1^DIAZI^ and PYR1^PIRI^ were tested in a protein-fragment complementation system based on split luciferase reconstitution with NLuc^N^-PYR1/NLuc^C^-HAB1 fusions in yeast (PYR1^DIAZI^, EC_50_ = 24 nM [12,50]; PYR1^PIRI^, EC_50_ = 19 nM [undef, 29]). **d**,**e**, PYR1^DIAZI^ and PYR1^PIRI^ are selective for their evolved target ligands. **d**, Y2H (top) and in vitro phosphatase inhibition assays (bottom) were used to profile receptor responses; the receptors no longer bind the native ligand ABA. Pirimiphos-methyl and diazinon were tested 20 nM, ABA, tested at 5,000 nM in Y2H assays. **e**, Characterization of receptor selectivity using a Z4-PYR1/VP64-ΔN-HAB1 gene activation circuit in the presence of the activating ligands shown (Supporting File 1 shows quantitative analyses of EC_50_ values). In all cases, the symbol represents the mean, and the error bars show 1 s.d. and may be smaller than the symbol. All data points represent the mean of triplicate data (*n* = 3), and error bars represent the standard deviation. Source data

Given the success of the PYR1 scaffold as a platform for cannabinoid sensing, we sought to explore the possibility of rapidly generating sensors for a second class of compounds. To do so, we screened single and multisite mutational libraries against various different organophosphates, an important class of toxic, nonselective acetylcholinesterase inhibitors that were among the first insecticides broadly used in the 20th century. Due to their effects on nontarget organisms, most organophosphates have been banned in the United States, and they present an ongoing environmental monitoring challenge. We screened PYR1 libraries against a panel of 10 organophosphates (diazinon, pirimiphos, dimethoate, chlorfenvinphos, parathion, disulfoton, azinphos, bromophos, malathion and monocrotophos), none of which activated wild-type PYR1 (Supplementary Fig. 1; see Supporting File 1 for library details). These screens yielded receptors for diazinon, pirimiphos, dimethoate and chlorfenvinphos at concentrations between 5 and 100 μM (Supporting File 3). In addition, we screened the new DSM library against the same set of 10 compounds and obtained receptors for seven of these (Supplementary Fig. 6). To improve receptor affinity, we used recombination-based mutagenesis for four organophosphate receptors (diazinon, pirimiphos, chlorfenvinphos and dimethoate) by shuffling hits against parent libraries. This approach was repeated four times, reducing the ligand concentration at each step to ultimately yield improved sensors for these two compounds (Fig. 4a,b). The diazinon-responsive variant PYR1^DIAZI^ is a heptuple mutant (E8G, V81Y, L87M, F108Y, M158V, F159G and A160V), and the pirimiphos-responsive variant PYR1^PIRI^ is an octuple mutant (K59R, S92M, N119S, S122Q, E130G, F159T, A160T and V174A). These optimized sensors were also immediately portable to the split luciferase system with low-nanomolar sensitivity (Fig. 4c). Together, these data demonstrate that the PYR1 ligand-binding pocket can mutate to accommodate organophosphate ligands and provide a general system for developing organophosphate sensors.

To address the selectivity profiles of the evolved organophosphate receptors, we characterized their cross-reactivity to target ligands, given the close structural similarity of diazinon and pirimiphos. Both HAB1 inhibition and Y2H assays showed that the evolved receptors are highly selective to their on-target ligands (Fig. 4d). PYR1^PIRI^ was activated by low-nanomolar concentrations of pirimiphos but required high-nanomolar to low-micromolar diazinon concentrations for activation above background levels. Similar results were observed with the PYR1^DIAZI^ receptor, and neither receptor was activated by ABA. In a strict test of specificity, we used our yeast transcription circuit to characterize the off-target responses of these engineered receptors to a panel of six chemically similar organophosphates. The PYR1^DIAZI^ EC_50_ to diazinon was greater than tenfold lower than all other molecules profiled (Fig. 4e). For example, PYR1^DIAZI^ responded with an EC_50_ of 1.1 μM to azinphos but with an EC_50_ of 43 nM to diazinon. Other off-target ligands responded with higher EC_50_ values and with lower activation levels (Supporting File 1). Similarly, the PYR1^DIAZI^ receptor showed good discrimination between its on-target ligand diazinon and other organophosphates when tested at 20 μM in the ELISA-format mode (Supplementary Fig. 7). Lastly, we examined how receptor selectivity changed over the evolutionary trajectory of the PYR1^DIAZI^ sensor. Off-target ligand responses remained weak throughout the evolutionary process and decreased as affinity increased (Supplementary Fig. 7). Collectively, these data show that improvements in affinity were not obtained at the cost of increased promiscuity and that high-affinity and high-selectivity can be evolved using the PYR1 scaffold.

**Extended Data Fig. 2:**
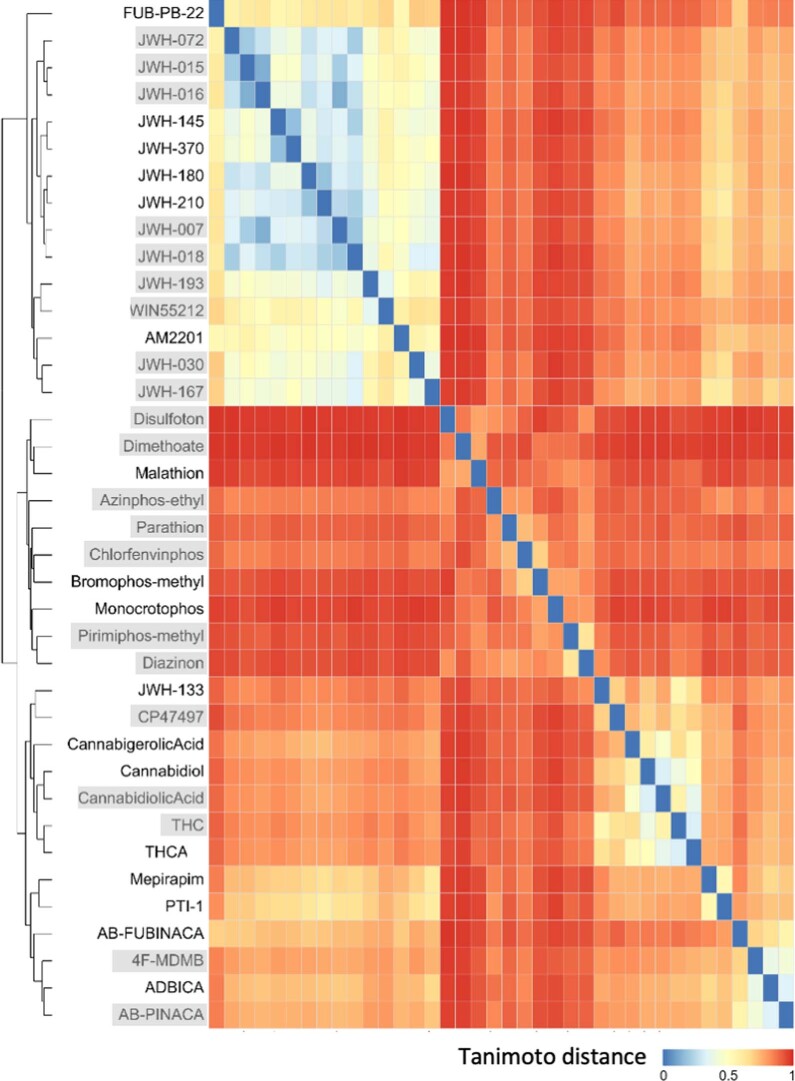
Chemicals subjected to sensor screens using PYR1 DSM library. The 38 chemicals screened in our manuscript were compared pairwise to compute a matrix of Tanimoto distance scores (shown in the heatmap), which was used for hierarchical clustering; blue = small distance (high similarity) and red = higher distance. Sensors were obtained for the 21 compounds in the shaded boxes.

**Extended Data Fig. 3:**
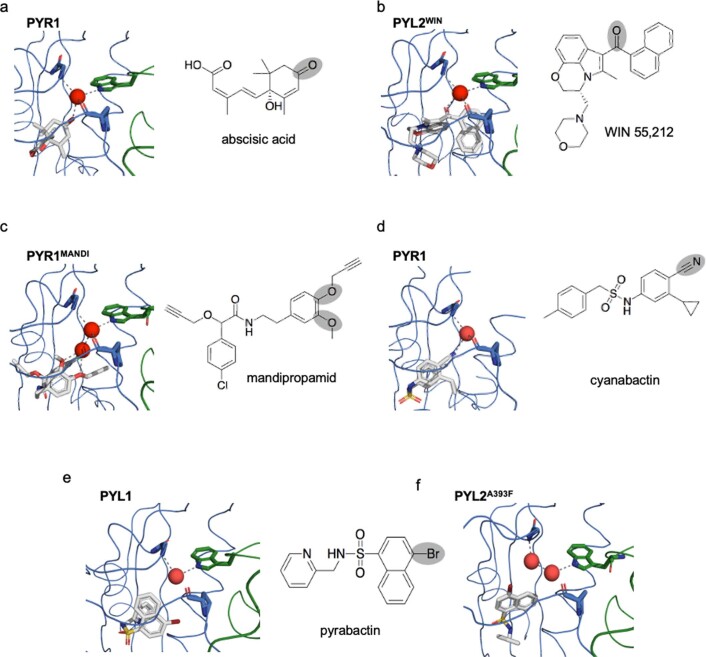
Diverse H-bond acceptors can stabilize activated PYR/PYL receptors. Diverse H-bond acceptors can stabilize activated PYR/PYL receptors. Structural comparisons of diverse receptor-ligand complexes with the receptor shown in blue and the complexed PP2C (HAB1) is shown in green. (**a**) In wild-type complexes, the PYR1/ABA/HAB1 complex is stabilized by a hydrogen-bond network coordinated by a central water molecule conserved across ligand-activated receptor X-ray structures (red). The conserved water stabilizes the ternary complex by making contacts to ABA’s cyclohexenone oxygen and PYR1’s P88 main-chain carbonyl oxygen, which serve as H-bond acceptors, and HAB1’s TRP385 indole HN and PYR1’s ARG116 main-chain NH, which serve as H-donors (PDB 3QN1). (**b**) In the engineered PYL2^WIN^ receptor, WIN 55,212’s naphthoylindole carbonyl oxygen interacts with the conserved water molecule to engage the TRP-lock, mimicking ABA’s carbonyl oxygen. (**c**) In the engineered PYR1^MANDI^ receptor, mandipropamid’s aryl-alkyl ether oxygens stabilizes the PYR1^MANDI^/mandipropamid/HAB1 complex via two bound waters that interact with ether oxygens (PDB 4WVO). (**d**) The rationally designed agonist cyanabactin stabilizes activated PYR1 via an arylnitrile H-bond acceptor that interacts with the conserved water (PDB 5UR6). (**e**, **f**) The agonist pyrabactin (with a weak C-Br H-bond acceptor) does not make appreciable contacts with bound water either in homologous PYL1 receptor nor in the engineered PYL2A393F. (e shows PDB ID 3NMT, native PYL1, PDB ID 3NMN is shown in f).

Using our designed PYR1-HAB1 system, we isolated sensors for 21 of the 38 compounds screened, which included a diverse set of ligands that fall into distinct chemotypes (Extended Data Fig. 2). Although structurally diverse, many of the ligands screened contain a carbonyl functional group that, as observed with WIN 55,212-2, can engage the Trp-lock to stabilize activated receptors (Fig. 2 and Extended Data Fig. 3). Prior work has shown that other H-bond acceptors (e.g., nitriles and others) can function in place of a carbonyl to activate both wild-type and engineered PYR1 receptors^11,29–31^ (Extended Data Fig. 3). In addition, our evolved PYR1^CP^ sensor recognizes a ligand lacking a C=O (Fig. 2). Thus, the chemical scope of ligands compatible with our system should be quite broad. However, even if there is a bias towards carbonyl-containing ligands, approximately one-third of natural products and drugs contain a carbonyl^32^ and provide a large set of ligands. When coupled to the high hit rates obtained with our DSM library, it should be possible to evolve molecular switches controlled by a large number of drugs, natural products or metabolites. Although many technologies for chemically regulated dimerization have been developed, our system is unique, because it empowers the design systems controlled by user-specified ligands, which is particularly useful when specific properties (such as low cost or low toxicity) are required in downstream applications. Thus, the PYR1/HAB1 system provides an easily reprogrammable chemical-induced dimerization module that will enable new applications in biotechnology, synthetic biology and medicine.

## Methods

### PYR1 library design

The design of the PYR1 pocket double-mutant library was performed with a combination of mutant stability analysis with the Rosetta molecular modeling suite and manual curation (Supplementary Information). The final library design includes mutations at 19 positions. Fifteen positions (59, 83, 89, 92, 94, 108, 117, 120, 122, 141, 159, 160, 163, 164 and 167) were allowed to mutate to all amino acids except cysteine or proline. Four positions (62, 81, 87 and 110) were restricted to smaller amino acid subsets.

### Construction of the PYR1 DSM library

The PYR1 DSM sub-libraries were constructed by NM as previously described^15^. Briefly, a library encoding pairs of mutations separated by at least eight amino acids was constructed by two sequential rounds of single-site NM using pooled primers (IDT). In parallel, an oligo pool (Agilent) containing primers encoding all pairs of proximate mutations (those separated by fewer than eight amino acids) was used in a single round of NM. The two libraries were then transferred to a two-hybrid vector by ligation and pooled to give the complete DSM library. See Supplementary Information for complete details of library construction, as well as libraries used for isolation of organophosphate sensors.

### Y2H screening of mutagenized PYR1 libraries

Selection experiments for mutant receptors that respond to new ligands were conducted as previously described^29,31^. Briefly, the PYR1 DSM mutant library was transformed into MAV99 harboring pACT-HAB1. Negative selections were conducted to remove receptors that bind HAB1 in a ligand-independent fashion (i.e., constitutive receptors) by growing the library on solid media containing 0.1% 5-fluoroorotic acid; the purged library was collected and used in subsequent selections for cells responsive to 30 µM cannabinoid ligands (purchased from Cayman Chemical as Drug Enforcement Administration-exempt preparations; Supplementary Fig. 2) on SD-Trp,-Leu,-Ura media. Colonies supporting uracil-independent growth at 30 °C were isolated after 3 days, retested to confirm ligand-dependent growth on SD-Trp,-Leu,-Ura plates with and without test chemical and then validated by β-galactosidase staining. Receptor ligand affinity optimization and new cannabinoid binders were obtained by generating and screening secondary shuffle (CB-S) libraries. To construct CB-S libraries, gene sequences of PYR1 cannabinoid-responsive variants were combined with the original PYR1 DSM library and the PYR1 SSM library in a ratio of 40/40/20, respectively, followed by recombinant-based mutagenesis, using nucleotide excision and exchange technology (NexT; see ref. ^34^). The resulting shuffled fragments were cloned into the Y2H pBD plasmid by restriction/ligation procedures. Ligation products were transformed into *Escherichia coli*, colonies were collected and plasmid DNA was extracted. In total, three independent shuffle libraries (CB-S1, CB-S2 and CB-S3) were generated at different stages of the optimization and rescreening process. CB-S1 and CB-S3 were screened with 10 to 0.1 μM ligand, depending on the sensitivity of the parental mutants. CB-S2 was screened with 0.05 to 0.025 μM ligand. The organophosphates screened (Supplementary Fig. 3 shows structures) were obtained from Sigma-Aldrich; screens of multisite mutagenized PYR1 libraries and affinity optimization details are provided in the Supplementary Methods.

### Ligand/receptor-mediated PP2C inhibition assays

Coding sequences of the cannabinoid sensors were cloned into the protein expression vector pET28a to encode 6×His-tag-receptor fusions. Constructs were sequenced and transformed into the BL21 (DE3) pLysS *E. coli* strain for heterologous expression with IPTG (1 mM), followed by purification, using affinity chromatography, as previously reported^35^. In vitro validation of evolved sensors was performed by using recombinant sensors and ΔN-HAB1 (HAB1 Δ1–178, ref. ^36^) proteins, as previously described^29^. Ligand/receptor-dependent inhibition of PP2C activity was performed essentially as previously reported using 25 nM ΔN-HAB1 with either 25 nM titrated PYR1^4F^ or 50 nM titrated PYR1^WIN^. Cannabinoid concentration curves ranged from 4 to 10,000 nM, and median inhibitory concentrations for PP2C inhibition were obtained via fluorescent measurement in the presence of 1 mM 4-methylumbelliferyl phosphate^37^.

### Cross-reaction tests for high-affinity cannabinoid and organophosphate sensors

High-affinity sensors were tested for cross-reaction using both Y2H and in vitro PP2C assays. PYR1^WIN^ and PYR1^4F^ sensors specificity was examined by X-gal staining using increasing amounts of WIN 55,212-2, 4F-MDMB-BUTINACA, AB-PINACA and JWH-072 (0, 50, 100, 200, 500 and 1,000 nM) and by in vitro PP2C inhibition assays (4–10,000 nM) with EC_50_ estimation for each receptor/ligand combination using nonlinear fitting with the [agonist] versus response function in GraphPad Prism 9. Similar methods were used to characterize PYR1^PIRI^ and PYR1^WIN^ sensor selectivity and sensitivity.

### Yeast transcriptional activation circuits

PYR1 variants were used to drive gene expression in an inducible genetic circuit by fusing a zinc-finger DNA-binding domain (Z4^38^) to the N terminus of PYR1 and the VP64 activation domain^39^ to the N terminus of ΔN-HAB1. The SV40 nuclear localization signal was also fused to the N terminus of ΔN-HAB1. A single 2µ plasmid was used to express Z4-PYR1 and SV40-VP64-ΔN-HAB1, whereas the GFP expression cassette (*Z4*_*4*_*-CYC1*_*core*_*-GFP-CYC1*_*term*_﻿) was integrated at the YPRCΔ15 site on chromosome XVI. GFP fluorescence induced by each circuit was measured 12 h after ligand addition to 1 ml cultures at 30 °C. Fluorescence was measured by flow cytometry. Briefly, 50 µl resuspended cells was transferred to a 96-well plate for analysis. The fluorescence intensity of each cell was measured using a BD Accuri C6 flow cytometer equipped with autoloading. The forward scatter, side scatter and GFP fluorescence (excitation/emission 488/533 nm) were recorded for a minimum of 10,000 events. Descriptions of the cloning procedures and methods used to create organophosphate- and cannabinoid-responsive transcriptional circuits are provided in the Supplementary Methods.

### In vivo split luciferase assays

To demonstrate a protein complementation output of PYR1-based cannabinoid sensors, the large and small fragments of split NanoLuc luciferase^24^ were fused to the N termini of PYR1 variants and ΔN-HAB1 and expressed from a 2μ plasmid. Ligand was added to yeast cultures 12 h before measuring cell luminescence. A sample of each culture was diluted to an OD_600_ of 0.2, 10 μl of which was transferred to a 96-well plate, the luciferase reagent mixture (Nano-Glo live cell substrate; Promega) diluted to a 1× concentration with DI water was added to the cell sample to a final volume of 200 μl. The relative luminescence signal was measured using a Synergy Neo2 Multi-Mode Microplate Reader in luminescence detection mode for 30 min after the addition of the reagents. The relative luminescence signal of each measured sample was taken as the average value of the time course once the signal had reached a plateau. Cloning methods and procedures for all split-luciferase systems are provided in Supplementary Methods.

### Construction of PYL2^4F^ and PYL2^WIN^

The PYL2 coding sequence cloned in the Y2H pBD vector was used as template to incorporate the corresponding PYR1^WIN^ and PYR1^4F^ homologous residues via site-directed mutagenesis using the QuikChange Lightning Multi Site-Directed mutagenesis kit (Agilent Technologies). Resulting clones PYL2^WIN^ (K64Q, F165A and V166I) and PYL2^4F^ (H119Q, Y124G, F165V and V166G) were sequence confirmed and incorporated into MAV99 harboring pACT-HAB1 and tested for ligand activation using uracil-independent growth and X-gal staining experiments. The same method was used to generate a PYL2^WIN^ version in a protein expression vector for crystallization studies.

### Expression, purification and characterization of variant HAB1s and PYLs

Except where noted, all proteins were expressed as N-terminal 6×His-MBP fusions primarily using the medium and method as described in ref. ^19^. For crystallographic structures, PYL2 and ΔN-HAB1^T+^ were expressed in *E. coli* and purified as described previously^29^. Assays for phosphatase activity and apparent T_m_, measured via thermal challenge using yeast surface display, were performed essentially as previously described^19^.

### HAB1 stabilization

ΔN-HAB1^T+^ and other stabilized dead-ΔN-HAB1 variants were designed as follows: loss of activity was encoded by either R199A, D243A or R199A and D204A; C186S, C274S mutations are known to improved redox stability^40,41^. Mutations conferring improved stability were identified by the Rosetta-based web server PROSS (all options default) using three separate HAB1 structures as starting points (Protein Data Bank (PDB) accession numbers 4WV0, 4DS8 and 3KB3)^42^. Consensus mutations shared between all structures were identified, yielding 22 potential mutations at 16 positions. These mutations were screened using empirical filters on distance from HAB1 interface and contact number^43^. Manual curation was used to identify the final design set of 11 mutations at 11 positions.

### ELISA-like assays

PYR1 was immobilized overnight in clear plates and blocked with KPL milk diluent in buffer CBS^19^ (CBSM). Detection was performed via binding a streptavidin-HRP conjugate to biotinylated ΔN-HAB1^T+^ followed by incubation with a commercial TMB reagent. For detection in biospecimens, well blocking was done with 1% BSA in buffer CBS (CBSB). The spiked biospecimens (that is human serum, saliva, or urine) were diluted 5-fold and mixed with the biotinylated ΔN-HAB1^T +^ in CBSB, before being added to the PYR1-modified wells. LOD was determined by the response data in the low linear range. The plot of absorbance (*A*_450_) versus log (target concentration) was fitted to linear regression and the resultant equation was used to calculate the target concentration that would yield the signal equal to (3× STD + blank), where STD is the standard deviation of blank.

### Crystallization

Purified protein was stored at −80 °C in a buffer containing 20 mM HEPES (pH 7.6), 50 mM sodium chloride, 10 mM dithiothreitol and 10% glycerol. Purified PYL2 and ΔN-HAB1^T+^ were mixed in 1:1.05 molar reaction and exchange into a buffer containing 20 mM HEPES (pH 7.6), 50 mM sodium chloride, 10 mM dithiothreitol, 5 mM magnesium chloride and 5% glycerol. The proteins were then concentrated to 15 mg ml^−1^ and incubated with a fivefold molar excess of (±)-WIN 55,212 (Cayman Chemical) for 30 min on ice. Crystallization of the PYL2:WIN:ΔN-HAB1^T+^ complex was conducted by sitting drop vapor diffusion at 19 °C. Drops were formed by mixing equal volumes of the purified PYL2:WIN:ΔN-HAB1^T+^ complex with well solution containing 100 mM Bis-Tris propane pH 6.5, 200 mM sodium bromide and 19% (w/v) PEG 3350. The resulting crystals were flash-frozen after passing through a cryoprotection solution consisting of well solution plus 20% glycerol. X-ray diffraction data for each complex were gathered from a single crystal. Diffraction data was collected at 100 K using the LS-CAT ID-21-F beamline at the Advanced Photon Source (Argonne National Laboratory). Diffraction data were indexed, integrated and scaled using the XDS software package^44^.

### Structure determination

The PYL2:WIN:ΔN-HAB1^T+^ complex structure was solved by molecular replacement using a PYL2:Quinabactin:HAB1 complex (PDB accession number 4LA7) devoid of ligand and water molecules as the search model to evaluate the initial phases. Phenix.AutoMR solved the initial phases and automatically built the majority of residues for both complexes^45^. The resulting models were completed through iterative rounds of manual model building in Coot and refinement with Phenix.refine^45^ using translational libration screw-motion (TLS) and individual atomic displacement parameters. A Phenix topology file for the (+)-WIN 55,512 ligand was generated using the PRODRG server (http://davapc1.bioch.dundee.ac.uk/cgi-bin/prodrg/)^46^. The geometry of the final structures was validated using Molprobity. Data collection and refinement statistics for the final PYL2:WIN 55,212-2:ΔN-HAB1^T+^ model are listed in Supplementary Table 5, and the coordinates for the structure have been deposited in the PDB with the accession number 7MWN.

### Reporting summary

Further information on research design is available in the Nature Research Reporting Summary linked to this article.

## Online content

Any methods, additional references, Nature Research reporting summaries, source data, extended data, supplementary information, acknowledgements, peer review information; details of author contributions and competing interests; and statements of data and code availability are available at 10.1038/s41587-022-01364-5.

## Supplementary information

Supplementary Information Supplementary Methods, Supporting File 1, Figures 1–7 and Table 1.

Reporting Summary

Supplementary Data 1 Sequence validation of mutagenic libraries and list of primers and plasmids used.

Supplementary Data 2 Sequences of cannabinoid sensors.

Supplementary Data 3 Sequences of organophosphate sensors.

## Data Availability

All source data for Figs. 3 and 4 are in the attached ‘Beltran_SourceData_Fig3.xlsx’ and ‘Beltran_SourceData_Fig4.xlsx’ workbooks. Sequences of all sensors described are listed in Supporting File 1. The following plasmids and libraries are available to noncommercial users through Addgene: pJS646 (Addgene ID 183175); pJS647 (Addgene ID 183176); PYR1 Double Mutant Library - proximal mutations (L007) (Addgene ID 183177); PYR1 Double Mutant Library - distant mutations (L008) (Addgene ID 183178). Raw sequencing reads for the PYR1 double-mutant library have been deposited in the Sequence Read Archive (BioProject accession number PRJNA830502). The coordinates for the structure have been deposited in the PDB with the accession number 7MWN. Source data are provided with this paper.

## Source data

Source Data Fig. 3 Statistical Source Data for Fig. 3.
Source Data Fig. 4 Statistical Source Data for Fig. 4.

## Extended data

are available for this paper at 10.1038/s41587-022-01364-5.

## Code availability

Custom code for evaluating the completeness of the PYR1 double-mutant library has been deposited in Zenodo (10.5281/zenodo.6493839). Source data are provided with this paper.
