# Supplementary material for: Rapid biosensor development using plant hormone receptors as reprogrammable scaffolds
Source: Nat Biotechnol. 2022 Jun 20;40(12):1855–61. doi: 10.1038/s41587-022-01364-5 (PMC9750858; doi:10.1038/s41587-022-01364-5)
Supplement: Supplementary file 1 — Supplementary Methods, Supporting File 1, Figures 1–7 and Table 1. [file 41587_2022_1364_MOESM1_ESM.pdf]

nature portfolio

https://doi.org/10.1038/s41587-022-01364-5

Supplementary information

Rapid biosensor development using plant hormone receptors as reprogrammable scaffolds

In the format provided by the authors and unedited

Rapid biosensor development using plant hormone receptors as reprogrammable scaffolds

Jesús Beltrán[1,2#], Paul J. Steiner[3,#], Matthew Bedewitz[3,#], Shuang Wei[4,#], Francis C. Peterson[5], Zongbo Li[6], Brigid E. Hughes[5], Zachary Hartley[1,2], Nicholas R. Robertson[7], Angélica V Medina-Cucurella[8], Zachary T. Baumer[3], Alison C. Leonard[3], Sang-Youl Park[1], Brian F. Volkman[5], Dmitri A. Nusinow[9], Wenwan Zhong[6], Ian Wheeldon[2,10,§], Sean R. Cutler[1,2,11,§], Timothy A. Whitehead[3,§]

[1] Department of Botany and Plant Sciences, University of California, Riverside, CA 92521, USA
[2] Institute for Integrative Genome Biology, University of California, Riverside, CA 92521, USA
[3] Department of Chemical and Biological Engineering, University of Colorado, Boulder, CO 80305, USA
[4] Department of Biochemistry, University of California, Riverside, CA 92521, USA
[5] Department of Biochemistry, Medical College of Wisconsin, Milwaukee, WI 53226, USA
[6] Department of Chemistry, University of California, Riverside, CA 92521, USA
[7] Department of Chemical and Environmental Engineering, University of California, Riverside, CA 92521, USA
[8] Department of Chemical Engineering and Materials Science, Michigan State University, East Lansing, MI 48824, USA
[9] Donald Danforth Plant Science Center, St. Louis, MO, 63132
[10] Department of Chemical and Environmental Engineering, University of California, Riverside, CA 92521, USA
[11] Center for Plant Cell Biology, University of California, Riverside, CA 92521, USA
# Authors contributed equally
§ Co-corresponding authors

This PDF file includes:

Extended Materials and Methods
Figs. S1 to S7
Table S1

Extended

Extended Materials and Methods

Plasmids

Plasmids were constructed using either NEBuilder HiFi DNA Assembly Master Mix (New England Biolabs), using a Q5 Site-Directed Mutagenesis Kit (New England Biolabs), or by nicking mutagenesis. All kits were used according to the manufacturer's instructions. Nicking mutagenesis was performed as previously described (Wrenbeck et al. 2016). All PCR products used in NEBuilder assemblies were fractionated by agarose gel electrophoresis and purified using a Monarch DNA Gel Extract Kit (New England Biolabs) or Zymo Research Gel Extract Kit. For Q5 Site-Directed Mutagenesis, PCR products were similarly gel extracted and 20 ng was used in a 5 µl KLD reaction. Plasmids were transformed into Mach1 chemically competent *E. coli* (Invitrogen). All primers, plasmids, and gblocks used are listed in **Supporting File 1**.

Plasmid pJS636 was constructed by NEBuilder HiFi assembly of pJS600 amplified with PJS-P2062/PJS-P2079, pJS600 amplified with PJS-P2080/PJS-P2081, and pJS600 amplified with PJS-P2082/PJS-P2063.

Plasmid pJS636-MBP was constructed by NEBuilder HiFi assembly of pJS636 amplified with pET29B+ backbone_fwd/pET29B+ backbone_rev, pMAL-C5G_ccDockerin amplified with met-asn-His-MBP-TEV_fwd/met-asn-His-MBP-TEV_rev, and pJS636 amplified with dN-HAB1_fwd/dN-HAB1_rev.

Plasmid pJS645 was constructed by NEBuilder HiFi assembly of pYTK084 amplified with PJS-P2112/PJS-P2113 and pBD-PYR1-BbvCI amplified with PJS-P2114/PJS-P2115.

Plasmid pJS646 was constructed by NEBuilder HiFi assembly of pBD-PYR1-BbvCI amplified with PJS-P2116/PJS-P2117, pBD-PYR1-BbvCI amplified with PJS-P2118/PJS-P2119, pYTK047 amplified with PJS-P2120/PJS-P2121, and pBD-PYR1-BbvCI amplified with PJS-P2122/PJS-P2123.

Plasmid pJS647 was constructed by Q5 Site-Directed Mutagenesis of pJS645 using primers PJS-P2124/PJS-P2125.

Plasmid pJS678 was constructed by single/multi-site nicking mutagenesis of pJS636-MBP with primer HABstarR199AD204A.

Plasmid pJS723 was constructed by NEBuilder HiFi assembly of pJS678 amplified with PJS-P2244/PJS-P2243, gBlock PJS-G0023, and gBlock PJS-G0024.

Plasmid pJS724 was constructed by NEBuilder HiFi assembly of pJS678 amplified with PJS-P2244/PJS-P2243, gBlock PJS-G0023 amplified by PJS-P2246/PJS-P2247, gBlock PJS-G0023 amplified by PJS-P2248/PJS-P2249, and gBlock PJS-G0024.

Plasmid pJS725 was constructed by NEBuilder HiFi assembly of pJS678 amplified with PJS-P2245/PJS-P2243, gBlock PJS-G0025, and gBlock PJS-G0026.

Plasmid pJS726 was constructed by NEBuilder HiFi assembly of pJS678 amplified with PJS-P2245/PJS-P2243, gBlock PJS-G0025 amplified by PJS-P2246/PJS-P2250, gBlock PJS-G0025 amplified by PJS-P2251/PJS-P2249, and gBlock PJS-G0026.

Plasmid pJS753 was constructed by multi-site nicking mutagenesis of pJS637 with primers PJS-P2335 and PJS-P2336.

Plasmid pJSS754 was constructed by multi-site nicking mutagenesis of pJS637 with primers PJS-P2336, PYR1-Y120H-CAT, and PYR1-A160G-GGT.

Plasmid pJS755 was constructed by NEBuilder HiFi assembly of pJS723 amplified with primers PJS-P2337/PJS-P2338 and pJS637 amplified with PJS-P2339/PJS-P2340.

Plasmid pJS756 was constructed by NEBuilder HiFi assembly of pJS723 amplified with primers PJS-P2337/PJS-P2338 and pJS624 amplified with PJS-P2339/PJS-P2340.

Plasmid pJS794 was constructed by NEBuilder HiFi assembly of pJS755 (template for all) amplified with PJS-P2337/PJS-P2338, PJS-P2339/PJS-P2340, PJS-P2401/PJS-P2395, PJS-P2334/PJS-P2402, and PJS-P2335/PJS-P2340.

Plasmid pJS796 was constructed by NEBuilder HiFi assembly of pJS755 (template for all) amplified with PJS-P2337/PJS-P2338, PJS-P2339/PJS-P2403, PJS-P2404/PJS-P2405, and PJS-P2406/PJS-P2340. Plasmid pJS799 was constructed by similar means.

Plasmid pSW004 was constructed by NEBuilder HiFi assembly in four steps. First, TEF1 promoter and TDH1 terminator amplified from the *S. cerevisiae* genome (primers SW019/SW020 and SW023/SW024), and PYR amplified with SW021/SW022 were assembled with the pIW15 plasmid backbone digested with Pfo1. Second, HAB1 amplified with SW017/SW018 was assembled with the first plasmid digested with Not1 and Spe1. Third, SV40-ZF4 (gblock SW-G001) was assembled with the plasmid digested with Not1. Finally, VP64 (gblock SW-G002) was assembled with the plasmid digested with AvrII. PYR1 variants ($PYR1^{4F}$ and $PYR1^{WIN}$) were amplified using SW021/SW022 and assembled with pSW004 digested with NheI and SacII. Plasmids expressing PYR1 organophosphate variants ($PYR1^{diazi}$ and $PYR1^{piri}$) were amplified using SW007/SW008 and assembled in a similar manner as $PYR1^{4F}$ and $PYR1^{WIN}$.

The GFP expression cassette was integrated into the YPRCΔ15 site on chromosome XVI of *S.cerevisiae* with plasmid pSW010, which was constructed from pIW156 that contains a HIS3 selection marker. NEBuilder HiFi assembly of fragments encoding 700 bp of upstream homology was amplified with SW025/SW026 from the *S. cerevisiae* genome, while the downstream homology region was amplified with SW035/SW036. Other fragments include, the Z4 DNA binding domain sequence fragment (gblock SW-G003), CYC1 core promoter amplified with SW027/SW028, and GFP amplified with SW029/SW030.

Split NanoLuc luciferase plasmid pSW143 was constructed from pSW004 in two steps with NEBuilder HiFi assembly. First, the SmBit-linker gblock (SW-G004) and pSW004 digested with AvrII and NheI were assembled. Second, LgBit-linker glock (SW-G005) and TEF1 promoter amplified with SW646/SW647 were assembled with the first step plasmid digested with EcorI and SacI. These manipulations create a system where the small NanoLuc protein fragment is fused to the N-terminus of HAB1, while the large protein fragment is fused to the N-terminus of PYR1. Plasmid expressing PYR1 variants ($PYR1^{4F}$ and $PYR1^{WIN}$) were amplified using SW422/SW423 and assembled with pSW143 digested with AvrII and NheI. Plasmids expressing organophoshate PYR1 variants ($PYR1^{DIAZI}$ and $PYR1^{PIRI}$) were amplified using SW043/SW044 and assembled in a similar manner as $PYR1^{4F}$ and $PYR1^{WIN}$.

PYR1 double mutant library design

Selection of residues to mutate

We examined PYR1 bound to ABA (PDB ID 3K90, chain A) and selected all residues either:

- with any atom within 5Å of ABA, or
- with any atom within 6Å of ABA and a Cα–Cβ vector not directed away from ABA.

After manual curation of the resulting list of positions, we removed the following residues:
- H60: Faces away from ABA and involved in PYR1 dimerization (Dupeux et al. 2011).
- F61: Outward facing and involved in PYR1 dimerization (Elzinga et al. 2019).
- R79: Appears to have only second-shell effects on ligand binding.
- P88: Conserved in the gate loop (Melcher et al. 2009).
- T91: Outward facing.
- H115: Conserved in the latch loop (Melcher et al. 2009).

Stability prediction in Rosetta

We first relaxed the PYR1 structure into the Rosetta score function using the following command:

```
~/Source/Rosetta/main/source/bin/relax.macosclangrelease \
-corrections::beta_nov16 \
-ex1 \
-ex2 \
-relax:constrain_relax_to_start_coords \
-relax:coord_constrain_sidechains \
-relax:ramp_constraints false \
-use_input_sc \
-flip_HNQ \
-no_optH false \
-s AtPYR1.pdb \
-out:no_nstruct_label \
-out:suffix _relaxed \
-out:file:o AtPYR1_relaxed.pdb \
-out:file:scorefile AtPYR1_relaxed_score.sc
```

The input file `AtPYR1.pdb` is chain A from PDB ID 3K90.

Next, we ran FilterScan in Rosetta to estimate the $\Delta\Delta G$ of all possible point mutations at each designed position. We used the following RosettaScripts XML:

```
<ROSETTASCRIPTS>

  <SCOREFXNS>
    <ScoreFunction name="f_soft" weights="beta_nov16_soft"/>
    <ScoreFunction name="f_hard" weights="beta_nov16"/>
  </SCOREFXNS>

  <RESIDUE_SELECTORS>
    <Index name="residues"
           resnums="%%pocket_residues%%"/>
  </RESIDUE_SELECTORS>

  <TASKOPERATIONS>
    <InitializeFromCommandline name="init"/>
    <OperateOnResidueSubset name="residue_subset"
                            selector="residues">
      <AddBehaviorRLT behavior="dummy"/>
    </OperateOnResidueSubset>
    <OperateOnResidueSubset name="residues_to_ignore">
```

```
      <Not selector="residues"/>
      <RestrictToRepackingRLT/>
    </OperateOnResidueSubset>
  </TASKOPERATIONS>

  <MOVE_MAP_FACTORIES>
  </MOVE_MAP_FACTORIES>

  <SIMPLE_METRICS>
    <TotalEnergyMetric name="energy_metric"
                       scorefxn="f_hard"/>
  </SIMPLE_METRICS>

  <MOVERS>
    <MinMover name="min_mover"
              scorefxn="f_hard"
              chi="true"
              bb="true"
              tolerance="0.001"
              max_iter="1000"/>
  </MOVERS>

  <FILTERS>
    <SimpleMetricFilter name="energy_filter"
                        metric="energy_metric"
                        comparison_type="lt"
                        cutoff="9999"
                        confidence="0"/>
    <Delta name="delta_energy"
           filter="energy_filter"
           upper="true"
           lower="false"
           range="-9999"/>
    <FilterScan name="scan"
                scorefxn="f_soft"
                task_operations="init,residue_subset,residues_to_ignore"
                relax_mover="min_mover"
                score_log_file="%%score_log_file%%"
                delta_filters="delta_energy"/>
  </FILTERS>

  <PROTOCOLS>
    <Add filter_name="scan"/>
  </PROTOCOLS>

  <OUTPUT/>

</ROSETTASCRIPTS>
```

And the following options:

```
-corrections::beta_nov16
-ex1
```

```
-ex2
-out:nooutput
-parser:protocol scan.xml
-parser:script_vars pocket_residues=59,62,81,83,87,89,92,94,108,110,117,120,122,141,159,160,163,164,167
-s AtPYR1_relaxed.pdb
```

Results are shown in **Supporting File 1**.

Final library design

To decrease the size of the library, we pruned mutations at positions where mutations appear particularly destabilizing or that appeared, by inspection of the structure, less likely to yield hits. Specifically, we removed mutations:

- At position 62 with $\Delta\Delta G > 7$ REU,
- At position 110 with $\Delta\Delta G > 5$ REU,
- At position 120 with $\Delta\Delta G > 5$ REU, and
- At position 160 with $\Delta\Delta G > 1$ REU.

In addition, we restricted mutations at position 87 to hydrophobic amino acids and glycine. These restrictions yielded the final library design given in **Supporting File 1**.

Construction of PYR1 mutant libraries

The PYR1 double mutant library (DSM) was constructed by nicking mutagenesis (Wrenbeck et al. 2016). The template used was pJS647, which contains the mutagenized portion of PYR1 flanked by BsaI sites and a BbvCI site for nicking. The full double mutant library was constructed in two parts. First, to construct a library of all distant mutations (those separated by more than eight amino acids), individual oligos encoding each mutation flanked by 24 bp of homology to the WT PYR1 sequence were pooled and used to construct a library of PYR1 single mutants (L004). This library was used as input to a second round of mutagenesis with the same pool of oligos, yielding a library of distant double mutants (L006). Second, to construct a library of all proximate mutations (those separated by fewer than eight amino acids), an oligo pool containing primers encoding all such proximate double mutations was synthesized (Agilent). This pool was used directly to perform nicking mutagenesis as previously described(Wrenbeck et al. 2016), yielding a library of all proximate double mutants (L005). After construction of libraries in pJS647, the PYR1 cassettes were transferred to pJS646 to reconstitute the full PYR1 Y2H vector. Briefly, library DNA and pJS646 were digested with BsaI-HFv2 (NEB) and fractionated by agarose gel electrophoresis. Bands corresponding to the 0.4 kb PYR1 cassettes (L005 and L006) and 6.6 kb vector (pJS646) were excised from the gel and DNA was recovered using a Monarch DNA Gel Extraction Kit (NEB). The library cassettes (7.6 ng) were ligated into the Y2H vector (82 ng) for 1 hour at 22 °C in a 10 µL reaction using T4 DNA ligase (NEB). The ligation reaction was purified using a Monarch PCR & DNA Cleanup Kit (NEB) and transformed into XL1-Blue electrocompetent *E. coli* (Agilent). Finally, the two libraries were pooled to give the full double-mutant library.

Three separate libraries were screened to isolate organophosphate sensors: a double mutant combination (DMC) library, a newly constructed PYR1-K59R-NNK library, and the computationally designed DSM library described above. The single site saturation K59R library was constructed using the PYR1-K59R mutant receptor template (Park et al. 2015) as three sub-libraries targeting positions 1-64, 65-128, or 129-191 using nicking mutagenesis (Wrenbeck et al. 2016) with degenerate NNK oligos spanning the entire coding sequence except R59 and H60. The PYR1-K59R-NNK library coverage was confirmed at greater than 99.9% by deep sequencing of libraries (library statistics are reported in **Supporting File 1)**. The PYR1 DMC library that we screened was previously constructed using oligo pool mutagenesis and (Medina-Cucurella et al. 2019) comprises combinations of 185 unique single mutations at 17 PYR1 positions (Park et al. 2015). The library shows 100% coverage of all 185 single mutants and 77% coverage (8,316/10,845) of the potential double mutants.

PYR1 library sequencing

Library coverage for the two final libraries (L007 and L008) was verified by deep sequencing. Briefly, 1 ng of library DNA was PCR amplified with primers PJS-P2165 and PJS-P2166 using 2xQ5 Master Mix (NEB) for twelve cycles with an annealing temperature of 64 °C and an extension time of 60 seconds. The product was purified with a Monarch PCR & DNA Cleanup Kit (NEB). Next, the libraries were indexed by PCR amplifying 1 ng of the purified product with Illumina primers RP1 and RPI1 (for L007) or RPI2 (for L008) using the same conditions. These reactions were purified using a Monarch PCR & DNA Cleanup Kit and then fractionated by agarose gel electrophoresis. The desired bands (503 bp) were excised and the DNA purified using a Monarch Gel Extraction Kit (NEB). Finally, the amplicons were further purified using AmpureXP beads according to the manufacturer's instructions. Deep sequencing was performed by the University of Colorado Boulder Biofrontiers Sequencing Core using an Illumina MiSeq with a 250 cycle paired end kit. Sequences were analyzed using custom scripts.

Yeast-two-hybrid screen of DSM library for cannabinoid sensors

Selection experiments for mutant receptors that respond to new ligands were conducted as previously described (Peterson et al. 2010 ; Park et al 2015). Briefly, the PYR1 DSM mutant library was transformed into MaV99 (pACT-HAB1) by electroporation (Lin et al. 2011) and screened on SD media supplemented with sorbitol (1 M). Negative selections were conducted to remove receptors that bind HAB1 in a ligand-independent fashion (*i.e.*, constitutive receptors) by growing the library on petri plates containing 0.1 % 5-fluoroorotic acid (5-FOA); the purged library was collected and used in subsequent selections for cells responsive to 30 μM of the synthetic cannabinoids (JWH-015, JWH-007, JWH-016, JWH-018, JWH-030, JWH-072, JWH-133, JWH-145, JWH-167, JWH-180, JWH-193, JWH-210, JWH-370, ADBICA, AM2201 8-quinolinyl carboxamide, Mepirapim, AB-FUBINACA, AB-PINACA, (±)-CP 47,497, (±)-WIN 55,212, FUB-PB-22, PTI-1, Cannabidiol and Δ9-THC) by growth on SD-Trp,-Leu,-Ura media (Sigma Aldrich); ~2 x 10^6 cells were plated per 100 x 15 mm petri dish. Colonies supporting uracil-independent growth at 30 °C were isolated after 3 days, re-tested to confirm ligand-dependent growth on SD-Trp,-Leu,-Ura plates with and with test chemical, and then validated presence/absence of test chemical in SD-Trp,-Leu, by ß-galactosidase staining using the chloroform-agarose overlay method. Plasmids from validated hits were isolated, propagated in *E. coli*, and sequenced. This process yielded double mutant hits for μM concentrations of JWH-015, JWH-016, JWH-018, JWH-030, JWH-072, WIN55,212 and CP 47,497. Additional cannabinoids 4F-MDMB-BUTINACA, cannabigerolic acid, cannabidiolic acid and THCA-A were screened with DSM shuffled libraries as described below.

Construction of secondary shuffled (CB-S) libraries

Plasmid DNAs of PYR1 cannabinoid-responsive variants were combined with the original PYR1 DSM library and the PYR1 K59R SSM "pocket" library (Park et al. 2015) in a ratio of 40/40/20 respectively, followed by recombinant-based mutagenesis, using nucleotide excision and exchange technology (NexT; (Müller et al. 2005)). Resulting shuffled fragments were cloned into the Y2H pBD plasmid by restriction/ligation procedures. Ligation products were transformed into E. coli, colonies collected, and plasmid DNAs extracted. In total, 4 independent optimization shuffle libraries (CB-S1, CB-S2, CB-S3 and CB-S4) were generated at different stages of the optimization and rescreening process as follow: CB-S1 included DNA templates from PYR1 double mutants F159A/A160I, F159A/V164H, Y120G/A160G, Y120H/A160G and Y120A/A160G. CB-S2 included DNA templates from PYR1 mutants K59N/Y120H/A160G, V81I/Y120G/A160G, K59N/Y120H/A160G, Y120G/M158I/A160G, K59Q/F159A/A160I, K59Q/F159A/A160I/Q169R, Y23H/D26G/K59Q/F159A/A160I. CB-S3 included DNA templates from PYR1 K59S/V83W/Y120G/F159A, Y120G/F159L/A160G, Y120G/F159V/A160G, Y120G/F159L/A160G, E4G/K59R/H115Q/Y120G with the PYR1 SSM and DSM libraries. CB-S4 included DNA templates from PYR1 mutants K59R/H115Q/Y120G/E141M, K59R/H115Q/Y120G/V164W/V190A and E4G/H115Q/Y120G/A160G, with the PYR1 SSM and DSM libraries.

Screening of (CB-S) libraries for improved affinity and more cannabinoid binders

All CB-S libraries (Except CB-S2) screenings for sensor/ligand affinity optimization were done in a range of concentrations (10-0.1 μM) depending on the sensitivity of the parental mutants tested at least otherwise stated. The CB-S1 library was screened on JWH-015, JWH-016, JWH-018, JWH-030, JWH-072, WIN 55,212. This enabled the identification of seven independent clones responding to cannabinoid concentrations as low as 50 nM for JWH-016 (PYR1 V81I/Y120G/A160G), JWH 072 (PYR1 K59N/Y120H/A160G), WIN 55,212 (PYR1 K59Q/F159A/A160I, PYR1 K59Q/F159A/A160I/Q169R, PYR1 Y23H/D26G/K59Q/F159A/A160I); to 100 nM for JWH-015 (PYR1 K59N/Y120H/A160G), and to 500 nM for JWH-018 (PYR1 Y120G/M158I/A160G).

The CB-S1 library was also screened on 30 μM of cannabinoids JWH-007, JWH-145, JWH-167, JWH-180, JWH-193, JWH-210, JWH-370, ABDICA, AM2201 8-quinolinyl carboxamide, Mepirapim, AB-FUBINACA, AB-PINACA, FUB-PB-22, PTI-1, cannabidiol, Δ9-THC, cannabigerolic acid, cannabidiolic acid and THCA-A. This experiment yielded sensors responding to μM concentrations of cannabinoids JWH-007 (PYR1 K59S/V83W/Y120G/F159A), JWH-167 (PYR1 Y120G, F159L, A160G), JWH-193 (PYR1 Y120G/F159V/A160G), AB-PINACA (PYR1 Y120G/F159L/A160G) and the phytocannabinoid Δ9-THC (E4G/H115Q/Y120G/A160G).

The CB-S2 library was screened on low (50 nM, 25 nM) concentrations of cannabinoids JWH-015, JWH-016, JWH-018, JWH-072, WIN 55,212 and JWH-030 (250 nM). This experiment enabled the identification of sensors with improved low nM affinity for JWH-015 (PYR1 K59N/Y120H/F159L/A160G), JWH-016 (PYR1 V81I/Y120G/F159L/A160G), JWH-072 (PYR1 E4G/K59N/Y120H/F159L/A160G) and WIN 55,212 (PYR1 E4G/Y23H /D26G/K59Q/F159A/A160I).

The CB-S3 library, was screened on decreasing concentrations (10, 1, then 0.1 μM) of cannabinoids JWH-007, JWH-167, JWH-193, AB-PINACA, (±)-CP 47,497, Δ9-THC, cannabidiol, cannabigerolic acid, cannabidiolic acid, THCA-A and 4F-MDMB-BUTINACA. This screening resulted in clones with improved affinity to JWH-007 (PYR1 V83W/Y120G/F159A/A160I), JWH-167 (PYR1 Y58H/V81M/V83F/H115Q/Y120G/F159L/A160G/D184G), JWH-193 (PYR1 L87M/A89V/E102K/Y120G/F159V/A160G), AB-PINACA (PYR1 E4G/H115Q/Y120G/F159V /A160G) , Δ9-THC (E4G/K59R/H115Q/120G) and to cannabinoids 4F-MDMB-BUTINACA (PYR1 H115Q/ Y120G/F159V/A160G) and CBDA (PYR1 K59R/H115Q/Y120G/E141M). To improve the affinities of the Δ9-THC and CBDA sensors, the CB-S4 library was screened on decreasing concentrations (10, 1, then 0.1 μM) of CBDA and Δ9-THC.

General expression and purification of variant HAB1s and PYRs

Proteins described in this work were expressed as N-terminal 6xHis-MBP fusions primarily using the medium and method as described in (Steiner et al., 2020), with following noted exceptions. Proteins used in the experiments described by Figure 3a were produced as in (Vaidya et al. 2019).

For catalytically dead HAB1 designs and all variants of PYR1, the supplemental $MnCl_2$ was excluded from ZY medium. Cell pellets were resuspended in Lysis Buffer at 4 mL g^{-1} wet cell weight and allowed to incubate for 15 minutes at bench temperature (20° C). Lysis buffer was comprised of 50% v/v B-PERII (ThermoFisher), 10% w/v glycerol, 50 mM HEPES, 200 mM KCl, 0.5 mM DTT, 5 mM TCEP, 10 mM imidazole, 15 mM $MgCl_2$, 0.2 U benzonase mL^{-1}, 0.1 mg mL^{-1} lysozyme, and 1 mM PMSF dissolved in DMSO to 100x, with the buffer adjusted to pH 8.0 with KOH as required. Additionally, 10 mM $MnCl_2$ was added to all buffers for preparation of catalytically active HAB1 variants, and for preparations of catalytically dead HAB1 variants when they were intended for direct comparison to catalytically active HAB1 designs. When $MnCl_2$ is included, a brown color may be observed. The clarified supernatant from 0.5 L of expression culture was incubated with gentle rocking for 1 hour with 5 mL of washed Ni-NTA resin (G-Biosciences) suspended in wash buffer supplemented with 5 mM ATP (JK Scientific Cat: 438666). The resin was allowed to settle by gravity on ice, and the supernatant was decanted into fritted columns. Once the resin was fully settled and the unbound fraction drained, 5 bed volumes of wash buffer were applied, comprised of 10% w/v glycerol, 50 mM HEPES, 500 mM KCl, 0.5 mM DTT, 5 mM TCEP, and 20 mM imidazole, at pH 8.0. Bound proteins were eluted with 2 column volumes of elution buffer (10% w/v glycerol, 50 mM HEPES, 200 mM KCl, 5 mM TCEP, and 500 mM imidazole, at pH 8.0).

On ice, ammonium sulfate powder was added to the elutions to 80% saturation as determined by the following tool (https://www.encorbio.com/protocols/AM-SO4.htm). These mixtures were incubated with gentle rocking for 20 minutes, followed by centrifugation for 10 minutes at 20,000*g*. The supernatant was removed by pipet, with a few minutes allowed for additional drainage of supernatant from tube and pellet. The pellets of proteins to be biotinylated were redissolved into biotinylation buffer, which for all MBP tagged proteins was pH 7.5, comprised of 10% w/v glycerol, 50 mM HEPES, 200 mM KCl, 5 mM TCEP. Proteins were desalted and quantified as previously described (Steiner et al 2019). All proteins to be biotinylated were either diluted or concentrated to 100 µM as required, and all MBP tagged proteins were biotinylated at a ratio of 20:1 at room temperature for 30 minutes. Biotinylation reactions were quenched as previously described and precipitated with saturated ammonium sulfate in 50 mM HEPES, 200 mM KCl, pH 8.0 to 80% saturation, with addition of DTT and TCEP as needed to maintain 1 mM of each. Suspensions were incubated on ice for 20 minutes, and pelleted by centrifugation for 10 minutes at 20,000*g*. Supernatants were removed and pellets were dissolved into desalting buffer comprised of 10% w/v glycerol, 50 mM HEPES, 200 mM KCl, 1 mM DTT, 5 mM TCEP at pH 8.0, desalted, and quantified again. Quantified proteins were precipitated with saturated ammonium sulfate with DTT and TCEP added to 1 mM each, incubated on ice, and pelleted by centrifugation at 10,000*g* for 15 minutes. The pellets were resuspended using saturated ammonium sulfate to a protein concentration of 100 µM, DTT and TCEP were added to 1 mM each, and the suspensions were stored at 4°C.

Proteins not getting biotinylated were redissolved directly into desalting buffer and were also quantified. These were precipitated by addition of ammonium sulfate powder to 80% saturation or greater. The suspensions were incubated on ice for 20 minutes and pelleted once again by centrifugation, this time at 10,000*g* for 15 minutes. The supernatants were removed, and the pellets resuspended using saturated ammonium sulfate to a protein concentration of 100 µM, DTT and TCEP were added to 1 mM each, and the suspensions were stored at 4°C.

GFP activation and fluorescence measurements

Transformed yeast cells were acquired by first inoculating 5 mL of liquid media and incubating in a shaker incubator overnight at 30 °C. Cells from the overnight culture were then used to inoculate a 50 mL culture with an initial cell density of 5 x 10^6 cells/mL. This culture was grown at 30 °C with 200 rpm shaking until ~$2x10^7$ cells/mL were produced, approximately 3 to 5 hours. Cells were harvested by centrifugation at 4000 rpm for 5 min, washed with 25 mL sterile, deionized water, resuspended in 1 mL of 100 mM LiAc, and transferred to a 1.5 mL tube, producing a cell suspension with approximately 10^9 cells/mL. The cell suspension was briefly mixed by vortexing and 100 µL was transferred into a clean 1.5 mL tube for each transformation. Cells were pelleted at 13000 rpm for 15 sec and the supernatant was removed. A transformation mixture of 240 µL PEG (50% w/v), 36 µL 1.0 M LiAc, 50 µL salmon sperm DNA (2.0 mg/ml), plasmid, and sterile water were added to the cell pellet and mixed by vortexing for 1 min (total volume of 360 µL). The mixed transformation solution was then heat shocked at 42 °C for 40 min. Post heat-shock, cells were recovered by centrifugation at 8000 rpm for 30 sec. Cells resuspended in 400 µL sterile water were plated on appropriate selection media, in this case agar-SD media without uracil. Plates were incubated at 30 °C until mature colonies were formed.

Three single transformants were picked from selection plates, inoculated in 2 mL of SD-Ura containing 2% glucose in 14 mL culture tubes. After 24 hours of growth at 30 °C in a shaker incubator (200 rpm), the OD600 of each sample was measured and cultures were back diluted to OD600 = 0.2 in fresh SD-Ura, the total volume of diluted culture was 9 mL. Each 9 mL culture was separated into 8 unique cultures by transferring 1 mL into 8 different wells of a 96 deep-well plate. Each well was used to evaluate the effect of a specific concentration of ligand. After all the cultures were transferred, 1 µL of stock ligand solution (each stock varying in concentration) was added to each well. The plate was sealed with an air-breathable polymer film and cultured with 1000 rpm shaking at 30 °C for 12 hours. Shaker humidity was maintained at 90%. Cells were harvested by centrifuge at 5000 g for 10 min, and after discarding the supernatant, the cells were suspended in 1 mL PBS buffer and centrifuged at 5000 g for 10 min. The cells were washed with 1 mL PBS buffer twice and

resuspended in 1 mL DI water for flow cytometry analysis. For flow cytometry analysis, 50 µL of resuspended cells were transferred to a 96-well plate with flat bottom, adding DI water up to final volume 200 µL. The fluorescence intensity of cells within each sample was measured using a BD Accuri C6 flow cytometer equipped with auto-loading from 96 well plates. The forward scatter, side scatter, and GFP fluorescence (Ex/Em 488/533 nm) were recorded for a minimum of 10,000 events.

Luminescence assays

A protein complementation output assay of evolved PYR-based sensors was carried out by NanoLuc split luciferase (19). The larger fragment, LgBit (or Luc^N), was fused to N-terminus of PYR1 variants, while the small fragment, SmBit (or Luc^C) was fused to N-terminus of ΔN-HAB1. Expression of both PYR1 variants and ΔN-HAB1 was accomplished from a single 2µ plasmid. gBlock encoding the luciferase fragments were purchased from Integrated DNA Technologies (IDT). This plasmid was then transformed into *S. cerevisiae* BY4742, cells were grown in 2 mL synthetic-defined medium without uracil (SD-U) containing 6.7 g L^{-1} yeast nitrogen base without amino acids (DB Difco®; Becton–Dickinson), and 1.92 g L^{-1} yeast synthetic drop-out medium supplements without uracil (Sigma-Aldrich). Ligand was added to yeast cultures 12 hour prior to measuring cell luminescence. Each culture was diluted to an OD600 of 0.2, 10 uL of which was transferred to a 96-well plate to make a final volume of 180 uL. 20 uL of the luciferase reagent mixture (Nano-Glo live cell substrate; Promega) was added to each well and gently mixed. The relative luminescence signal (RLU) was immediately measured continuously in a Synergy™ Neo2 Multi-Mode Microplate Reader over 30 minutes. The average value of RLU of each measured sample was taken once the measurements had reached a stable value. Three single transformants were picked from selection plates as done for the fluorescent measurements.

Modifications to the general expression and purification protocol for PYR^{4F}

Prior to centrifugation of cell pellets, flasks of cells to be harvested were incubated on ice for 30 minutes. All further steps were performed on ice, using buffers, tubes, and columns pre-incubated on ice, and the lysis buffer was further supplemented to 2.0 U benzonase mL^{-1}, 1.0 mg mL^{-1} lysozyme. Lysis was allowed to proceed for 2 hours, rather than 15 minutes.

Determination of apparent T_m for HAB1 variants

Citrate-buffered saline was first prepared (CBS: 20 mM sodium citrate, 147 mM NaCl, 4.5 mM KCl). A portion of this CBS was removed and this aliquot was made into CBS^{+++} by addition of freshly dissolved DTT to 1 mM, TCEP pH 8.0 to 1 mM, and $MnCl_2$ to 10 mM followed by pH adjustment to 8.0 using 1 M sodium hydroxide. CBS^{+++} was then sterile filtered and placed on ice. A further portion of CBS was removed and the above reagents were added in addition to bovine serum albumin at 0.2% w/v to produce $CBSFF^{+++}$, followed by adjustment of pH to 8.0 with 1 M sodium hydroxide and the solution was again sterile filtered and placed on ice. The remainder (and bulk) of the CBS was used to produce CBSF by addition of 0.1% w/v BSA with adjustment of pH to 8.0 using 1 M sodium hydroxide, and this solution was also sterile filtered, and 25 mL were retained at room temperature, with the bulk of the solution transferred to ice.

Determination of apparent T_m was performed using thermal melt. 130 µL of the ice-cold ammonium sulfate precipitates of HAB variants MBP-636 (“WT”), ΔN-$HAB1^{T+}$, MBP-724, MBP-725, and MBP-726 were pelleted in a microcentrifuge for 5 minutes at 17,000*g*. The supernatant was removed by pipette, discarded, and the pellet resuspended in an equivalent volume of ice-cold CBS^{+++}. Zeba™ spin desalting columns (Thermo) were equilibrated with CBS^{+++} and the resuspended HABs were desalted and placed on ice. Each HAB was diluted to 400 nM concentration in CBS^{+++} and aliquoted into PCR tubes at a 50 µL volume. These tubes were distributed into the blocks of a pair of Eppendorf Mastercycler X50i thermocyclers programmed for a thermal gradient as follows (in °C): (Machine #1 Gradient 30.0 to 60.0 – 30.0, 35.4, 39.3, 46.8, 50.6, 54.5, 60.0, Machine #2 Gradient 65.0 to 95.0 – 65.0, 70.4). The HAB aliquots were held at these temperatures for 30 minutes, followed by cooling to 4 °C in the thermocycler blocks. Once the aliquots had cooled, they were transferred to racks on ice and 50 µL of $CBSFF^{+++}$ was added, such that each HAB was now at a concentration of 200 nM (10X) in

CBSF^{+++}. The heat treated HAB aliquots were left on ice until the remainder of the labeling reactions were set up, about 1 hour. Labeling reactions were performed, measured, and analyzed as described previously (Steiner et al. 2020). Anti-cmyc FITC (Miltenyi Biotec, part number 130-116-653) was used in these experiments with a dilution of 1:50.

Reconstitution of the PYR/HAB based CID system in a plate-based ELISA

Unconjugated 6xHis-MBP-PYR1 variants were immobilized to Microlon clear plates (655081) using 100 µL of CBS without pH modification, at a protein concentration of 100 µg mL^{-1}, or 1.57 µM at 4°C overnight. Wells assigned as no PYR controls received only CBS. Plates were sealed using Microseal B adhesive sealers (BioRad MSB-1001). The following day, the solutions were decanted by flicking and all wells were washed twice with 300 µL of CBS. Blocking was performed using 300 µL of KPL milk diluent/concentrate (SeraCare 5140-0011) diluted 1:20 according to manufacturer's protocol, hereafter referred to as CBSM, incubated at room temperature for 2 hours, during which time all other components of the assays were diluted and assembled in 96 well PCR plates (Biorad HSP9601). Binding reactions were assembled from 88 µL of CBSM, 2 µL of 50x concentrate of ligand, dissolved in anhydrous ethanol for ABA, or anhydrous DMSO for all other compounds, with 10 µL of HAB to be added. Blocked plates were decanted by flicking and were washed twice with 300 µL of CBSM, followed by vigorous tamping on a pad of paper towels. The sole exception to the pre-assembly was biotinylated ΔN-HAB1^{T+}, where 130 µL of ammonium sulfate precipitate was pelleted at 17,000*g* for 10 minutes, and the supernatant removed. The pellet was stored on ice until the blocked and washed plates were ready to receive the binding reactions. ΔN-HAB1^{T+} was exchanged into CBSM with 1 mM DTT and 1 mM TCEP (CBSM++) using Zeba™ spin desalting columns (Thermo) were equilibrated with CBSM++. ΔN-HAB1^{T+} was diluted to 10x the final binding reaction concentration and was immediately added to the pre-assembled assays in PCR plates, which were mixed by pipetting and transferred to the washed, blocked Microlon plates. Microlon plates were transferred to a plate shaker (Heidolph Titramax 1000) for 30 minutes at room temperature with shaking at 800 rpm. Plates were decanted by flicking and were washed three times with 300 µL of CBSM, followed by vigorous tamping on a pad of paper towels. Detection was enabled by addition of 100 µL of streptavidin-horseradish peroxidase (Thermo, SA10001), diluted 1:25,000 into CBSM, incubated in the above plate shaker for 10 minutes. This was decanted by flicking and washed three times with 300 µL of CBSM and by vigorous tamping on a pad of paper towels. Bound HAB was detected through addition of 50 µL 1-Step™ Ultra TMB-ELISA Substrate Solution (Thermo, 34028), incubated in the above plate shaker for 15 minutes (PYR1$^{M\ (N90S/H60P)}$ with ABA, PYR1$^{WIN\text{-}M}$ and PYR1$^{4F\text{-}M}$ in specificity assays to avoid overdevelopment of positive control), or for 30 minutes (PYR1$^{WIN\text{-}M}$ and PYR1$^{4F\text{-}M}$ under ligand titrations; PYR1$^{DIAZI\text{-}M}$ was used for substrate specificity screening). TMB development was quenched by addition of 50 µL of 2M sulfuric acid and plates were read at 450 nm using a Biotek Synergy H1 Hybrid multimode plate reader. Estimates of $K_{d,eff}$ were performed using Graphpad Prism 8.4.3 using the specific binding with Hill slope nonlinear regression function. Best fits for K_{d1} and K_{d2} were determined from global nonlinear fitting of the ELISA data using a custom script written in MatLab 2019b.

Detection in biospecimens followed the assay procedure above with minor modifications. Plate blocking was conducted in CBS supplemented with 1% bovine serum albumin (BSA; CBSB). CBSB was also used for washing the 96-well plate in between incubation steps. 20 µL of serum, saliva, or urine (Innovative Research, Inc.) spiked with 2 µL (+)-WIN 55,212-2 (mesylate) in DMSO was mixed with 10 µL ΔN-HAB1T+ prepared in CBSB plus 1 mM DTT and 1 mM TCEP and diluted with CBSB to reach a final volume of 100 µL before being added into the blocked wells. In the final assay solution, the concentration of (+)-WIN 55,212-2 (mesylate) was 0, 1 pM, 10 pM, 100 pM, 1 nM, 10 nM, 100 nM, and 1 µM; and the biospecimen was diluted by 5-fold. Streptavidin-horseradish peroxidase diluted 1:20,000 in CBSB was added to enable detection. Signal development was done through the addition of 100 µL of the TMB-ELISA Substrate and quenched by 100 µL of 2M sulfuric acid. LOD was determined by the response data in the low linear range and is reported as five times the value to account for the diluted biospecimen. The plot of absorbance (A450) vs. Log (target concentration) was fitted to linear regression and the resultant equation was used to calculate the target concentration that would yield the signal equal to (3* STD+blank), where STD is the standard deviation of blank.

Crystallization

PYL2 and ΔN-HAB1^{T+} were expressed in *E. coli* and purified as described previously (Vaidya et al. 2019). Purified protein was stored at -80 °C in a buffer containing 20 mM HEPES (pH 7.6), 50 mM sodium chloride, 10 mM DTT and 10% glycerol. Purified PYL2 and ΔN-HAB1^{T+} were mixed in 1:1.05 molar reaction and exchange into a buffer containing 20 mM HEPES (pH 7.6), 50 mM sodium chloride, 10 mM dithiothreitol, 5 mM magnesium chloride and 5% glycerol. The proteins were then concentrated to 15 mg/mL and incubated with a 5-fold molar excess of (±)-WIN 55,512 (Cayman Chemical) for 30 minutes on ice. Crystallization of the PYL2:WIN:ΔN-HAB1^{T+} complex was conducted by sitting drop vapor diffusion at 19 °C. Drops were formed by mixing equal volumes of the purified PYL2:WIN:ΔN-HAB1^{T+} complex with well solution containing 100 mM bis-tris propane pH 6.5, 200 mM sodium bromide and 19% (w/v) PEG 3,350. The resulting crystals were flash-frozen after passing through a cryoprotection solution consisting of well solution plus 20% glycerol. X-ray diffraction data for each complex were gathered from a single crystal. Diffraction data was collected at 100 K using the LS-CAT ID-21-F beamline at the Advanced Photon Source (Argonne National Labs, Lemont, IL). Diffraction data were indexed, integrated, and scaled using the XDS software package (Kabsch 2010).

Structure determination

The PYL2:WIN:ΔN-HAB1^{T+} complex structure was solved by molecular replacement using a PYL2:Quinabactin:Hab1 complex (PDB ID 4LA7) devoid of ligand and water molecules as the search model to evaluate the initial phases. Phenix.AutoMR (Adams et al. 2010) solved the initial phases and automatically built the majority of residues for both complexes. The resulting models were completed through iterative rounds of manual model building in Coot (Emsley and Cowtan 2004) and refinement with Phenix.refine using translational libration screw-motion (TLS) and individual atomic displacement parameters. A Phenix topology file for the (+)–WIN 55,512-2 ligand was generated using the PRODRG server (http://davapc1.bioch.dundee.ac.uk/cgi-bin/prodrg/)(Schüttelkopf and van Aalten 2004). Geometry of the final structures were validated using Molprobity (Davis et al. 2007). Data collection and refinement statistics for the final PYL2:WIN:ΔN-HAB1^{T+} model are listed in Supplementary Table S1 and the coordinates for the structure deposited in the Protein Data Bank, PDB ID 7MWN.

Screens for organophosphate sensors

MAV99 harboring either the K59R-NNK, DMC, or the DSM libraries were subjected to negative selections to remove constitutively active receptors by plating onto synthetic dextrose (SD) medium lacking leucine and tryptophan, and supplemented with 0.1% FOA (SD,-Leu,-Trp,+FOA). Surviving cells were collected and used in subsequent positive selections for ligand-responsive receptors on SD,-Leu,-Trp,-Ura media supplemented with a specific test chemical (100 µM) for responsiveness to any of the 10-member panel of organophosphate compounds: diazinon, pirimiphos-methyl, azinphos-ethyl, dimethoate, chlorfenvinphos, disulfoton, parathion-methyl, bromophos-methyl, malathion, and monocrotophos; all compounds were purchased form (Sigma-Aldrich). Isolated colonies were tested by regrowth on ligand-containing media and by dose response using X-gal staining and Sanger sequenced. Hits isolated from the DMC library were used in subsequent optimization efforts.

Organophosphate sensor optimization

Recombination-based mutagenesis of hit receptors was used to generate subsequent molecular diversity for selection-based optimization experiments, using the same general strategy across all scaffolds utilized. In the case of the PYR1-derived organophosphate sensors, plasmids for ligand-responsive mutants were diversified for subsequent optimization by recombination with one another, the parental DMC, and previously described PYR1 and PYR1^{K59R} SSM libraries that harboring saturating mutations in 39 and 25 sites, respectively (Park et al. 2015; Mosquna et al. 2011). These DNAs were mixed in a 40:20:20:20 ratio (hit mutants/DMC/SSM/K59R-SSM), and the PYR1 coding sequence PCR amplified from the pool, and subjected to recombination-based mutagenesis using nucleotide excision and exchange technology (NexT)(Müller et al. 2005). The resulting shuffled DNA fragments were cloned into pBD, transformed into *E. coli*, colonies collected, and plasmids extracted. In total, four optimization libraries (OP-S1, OP-S2, OP-S3, OP-S4) were constructed throughout the

optimization process by adding newly isolated hit receptors at each stage to the DMC/SSM/K59R-SSM DNA library pool used for NeXT mutagenesis. The initial organophosphate receptors for diazinon, pirimiphos-methyl, chlorfenvinphos, and dimethoate were combined to create OP-S1, which was constructed using the following PYR1 mutants: K59R/M158T, K59R/M158V, K59R/F159T, K59R/F159C, K59R/F159I, K59R/Y120A, Y120A/F159L, Y120A/F159T, L87P/Y120A, Y120A/F159T, L87M/Y159G, F108Y/F159G, K59R/F108A, K59R/F159L, K59R/I135R/ T162W and N167G. OP-S2 was constructed using PYR1-S16P/S29G/Q36R/K59R/S92M/F159 and PYR1-F108Y/D154G/ F159G/A160V. The OP-S3 library used the following mutants: PYR1-K59R/S92M/F159T/V174A and PYR1-L87M/F108Y/F159G/A160V. Finally, OP-S4 used PYR1-K59R/S92M/S122Q/E130G/ F159T/V174A and PYR1 V81Y/L87M/F108Y/F159G/A160V.

FIGURES

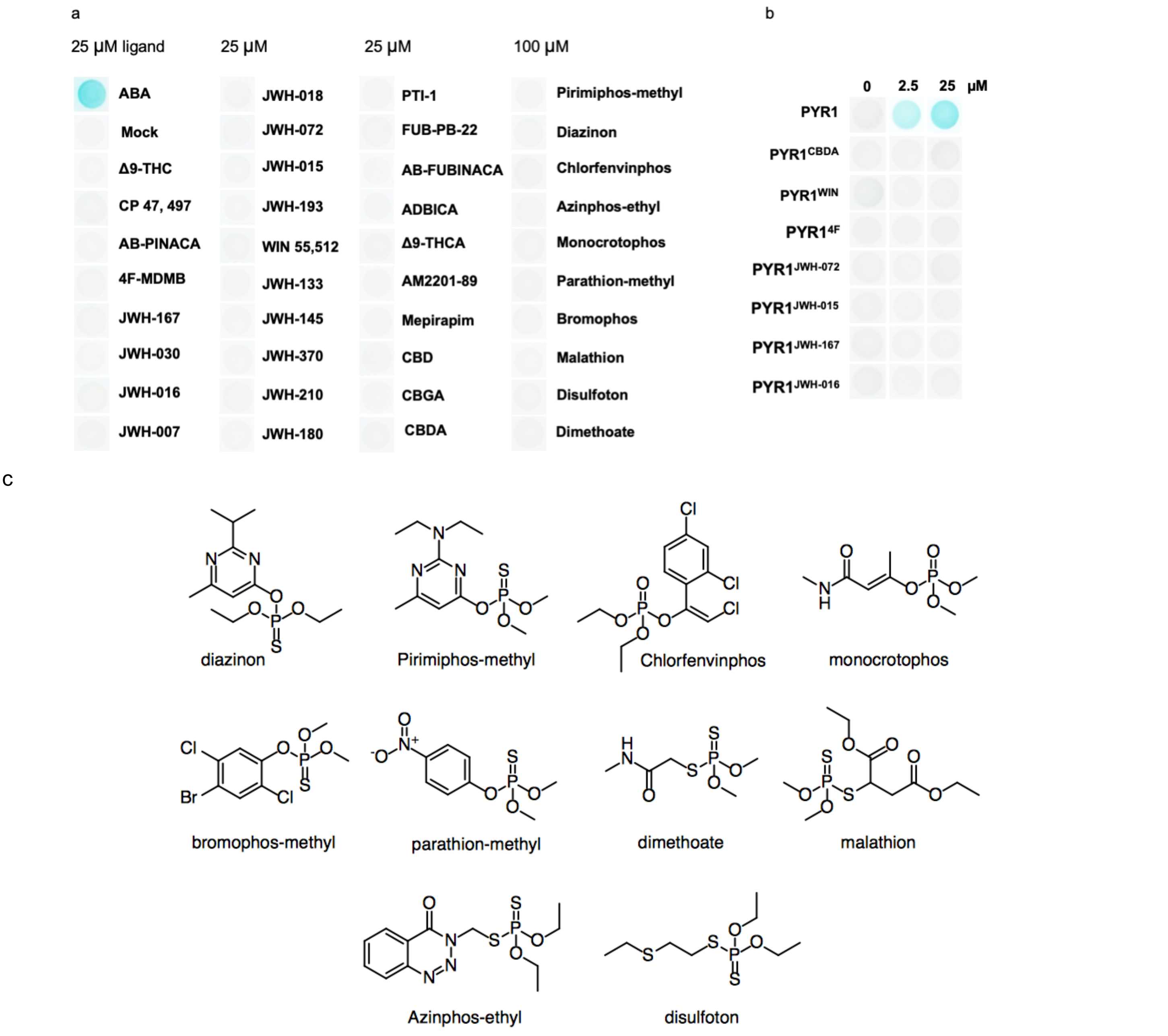

Supplemental Figure S1. Wild type and engineered PYR1 responses to diverse ligands.

(a) Wild type PYR1 does not respond to the structurally diverse cannabinoid and organophosphate ligands tested in this manuscript. Wild type PYR1 was tested for responsiveness to its native ligand ABA (25 μM) or the 28 cannabinoids (25 μM) and 10 organophosphates (100 μM) screened in this work; tests were conducted using the PYR1/HAB1 Y2H assay (see methods).

(b) PYR1-derived cannabinoid sensors do not respond to ABA. PYR1 and multiple evolved sensors were tested for responsiveness to ABA (0, 2.5, and 25 μM); the ABA non-responsiveness of PYR1^{DIAZI} and PYR1^{PIRI} are shown in the main text Figure 4d.

(c) Organophosphates screened in this work. Cannabinoids screened are shown in Fig S1.

a

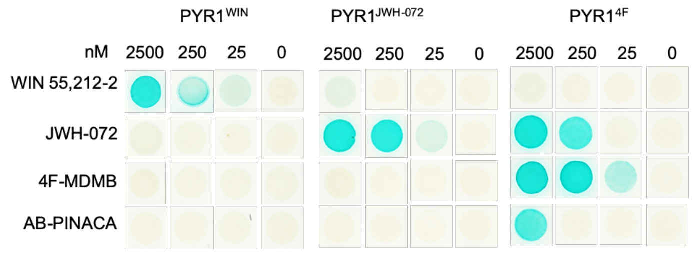

b

Evolved Sensor	E4	Y23	D26	K59	V83	H115	Y120	E141	F159	A160	V164
PYR1^{CBDA}				R		Q	G	M			W
PYR1$^{\Delta 9\text{-}THC}$				R		Q	G				
PYR1$^{AB\text{-}PINACA}$						Q	G		V	G	
PYR1^{4F}						Q	G		V	G	
PYR1$^{JWH\text{-}167}$					F	Q	G		L	G	
PYR1$^{JWH\text{-}016}$							G		L	G	
PYR1$^{JWH\text{-}007}$					W		G		A	I	
PYR1$^{JWH\text{-}018}$							G			G	
PYR1$^{JWH\text{-}193}$							G		V	G	
PYR1^{CP}				A	L			F			
PYR1^{WIN}	G	H	G	Q					A	I	

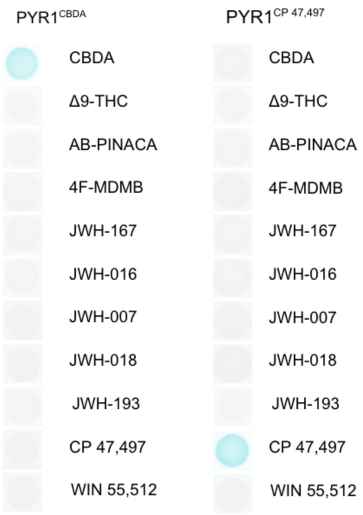

c

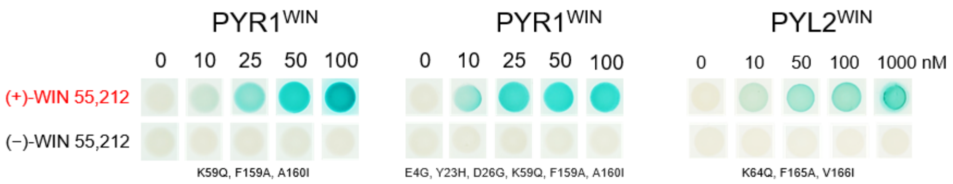

d

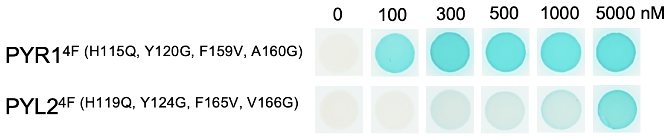

Supplemental Figure S2.

(a) Sensor cross-reactivity of PYR1-based sensors for synthetic cannabinoids. We note that a subset of the staining data shown in this figure for PYR1^{4F} was used in the main text Fig. 2b to illustrate the PYR1^{4F} sensor response.

(b) The evolved PYR1^{CBDA} and PYR1$^{CP\ 47,497}$ sensors selectively recognize their target ligands. (**a**) The high-affinity CBDA sensor PYR1^{CBDA} shares numerous mutations in common with other evolved cannabinoid sensors, including the space-opening pocket mutation Y120G (blue).

(c) PYR1^{CBDA} selectively responds to CBDA in spite of the common volume-expanding Y120G mutation shared between PYR1^{CBDA} and other evolved cannabinoid sensors; all ligands tested at 25 μM. (**c**) PYR1$^{CP\ 47,497}$ selectively responds to CP 47,497 and has a divergent ligand-binding pocket as does PYR1^{WIN} (see main text Figure 2).

(d) Sensitivity of the PYR1 and PYL2 variants of the WIN 55,212-2 and 4F-MDMB-BINACA sensors. The WIN 55,212-2 sensors are selectivity toward the (+)-stereoisomer; we note that a subset of the staining data shown in this figure for (+)-WIN 55,212-2 was used in the main text Figure 2b to illustrate the PYR1^{WIN} sensor response.

a

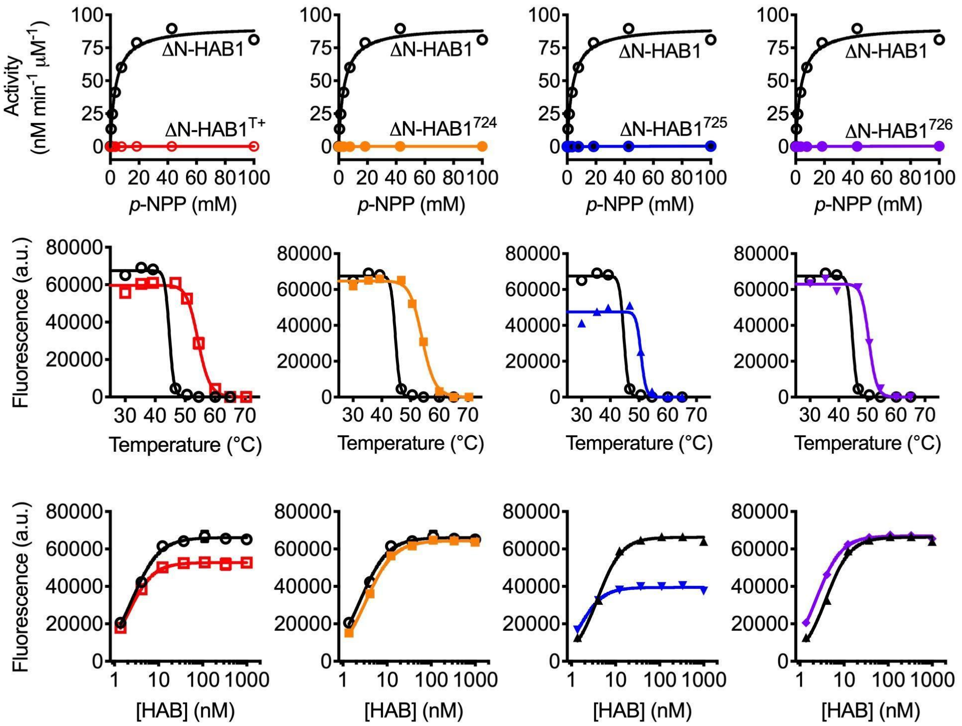

Activity (nM min-1 μM-1)
ΔN-HAB1
ΔN-HAB1T+
ΔN-HAB1724
ΔN-HAB1725
ΔN-HAB1726
p-NPP (mM)
Fluorescence (a.u.)
Temperature (°C)
[HAB] (nM)

b

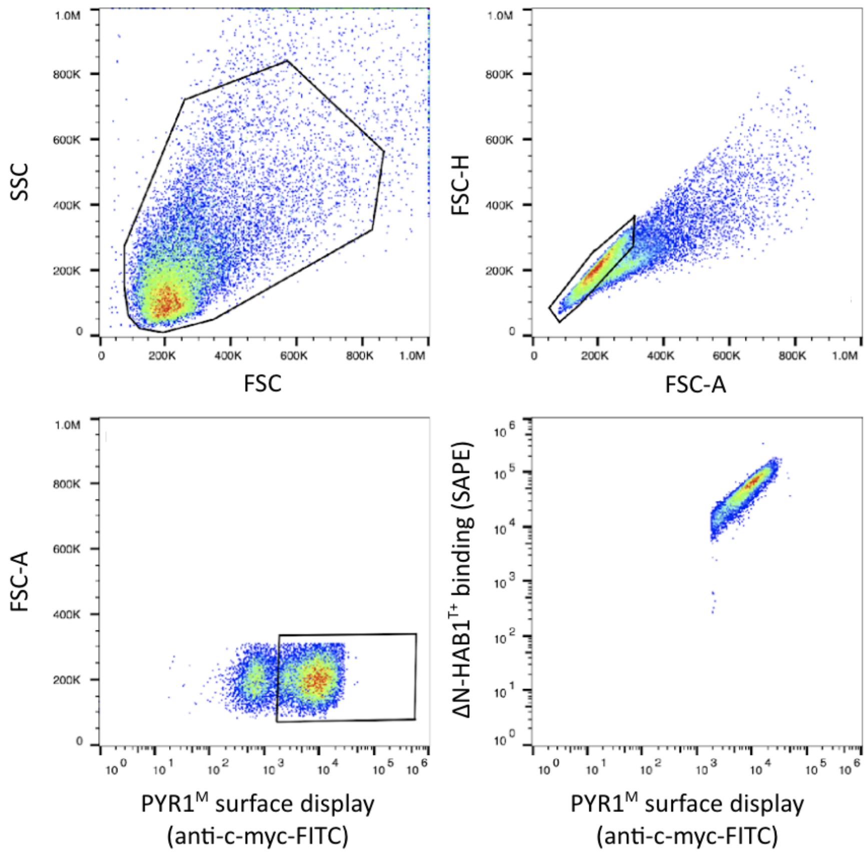

SSC
FSC
FSC-H
FSC-A
ΔN-HAB1T+ binding (SAPE)
PYR1M surface display
(anti-c-myc-FITC)

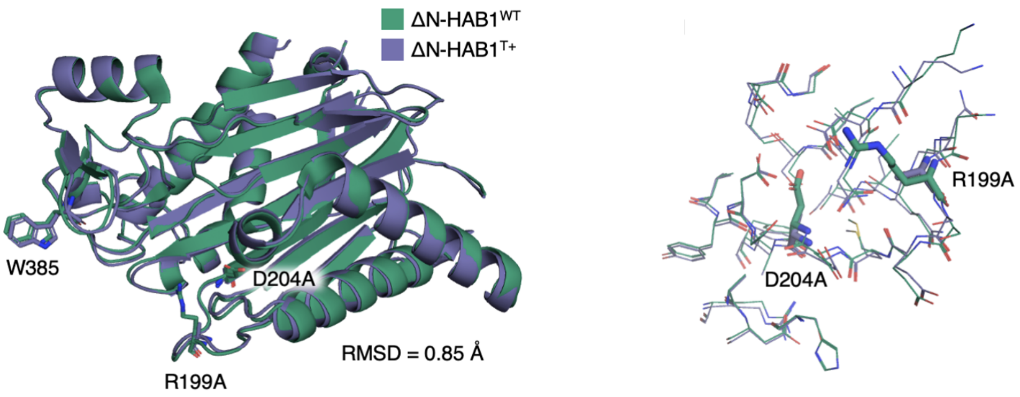

Supplemental Figure S3.

(a) Assessment of stabilized, inactivated 6xHis-MBP-ΔN-HAB1 variants. Experiments were repeated on two separate days using independently generated batches of protein. Error bars indicate 1 SD of 3 measurements. Sets of mutations for each variant are noted in **Supporting File 1**. The first row shows Michaelis-Menten enzyme kinetics for 6xHis-MBP-ΔN-HAB1 variants using p-NPP. The second row shows determination of apparent Tm for 6xHis-MBP-ΔN-HAB1 variants using YSD of PYR1^{M}, in the presence of 500 nM ABA and 20 nM 6xHis-MBP-ΔN-HAB1 variant. The third row shows YSD titrations of noted HAB1 variant against PYR1^{M} in the presence of 500 nM ABA. Fluorescence here refers to streptavidin-phycoerythrin.

(b) Gating strategy for analysis of data shown in part (a). The fluorescence (a.u.) reported in part (a) is the gated ΔN-HAB1^{T+} binding as measured by streptavidin-phycoerythrin (SAPE).

(c) The structure of ΔN-HAB1^{T+} is unchanged compared to ΔN-HAB1^{WT}. (**a)** Alignment of ΔN-HAB1^{WT} (PDBID: 3QN1) to ΔN-HAB1^{T+}. All-atom RMSD is 0.85 Å. The tryptophan latch W385 and two mutated active site residues R199A/D204A are shown as sticks. (**b)** Superimposed active sites of the two proteins. Individual rotamers are essentially unchanged.

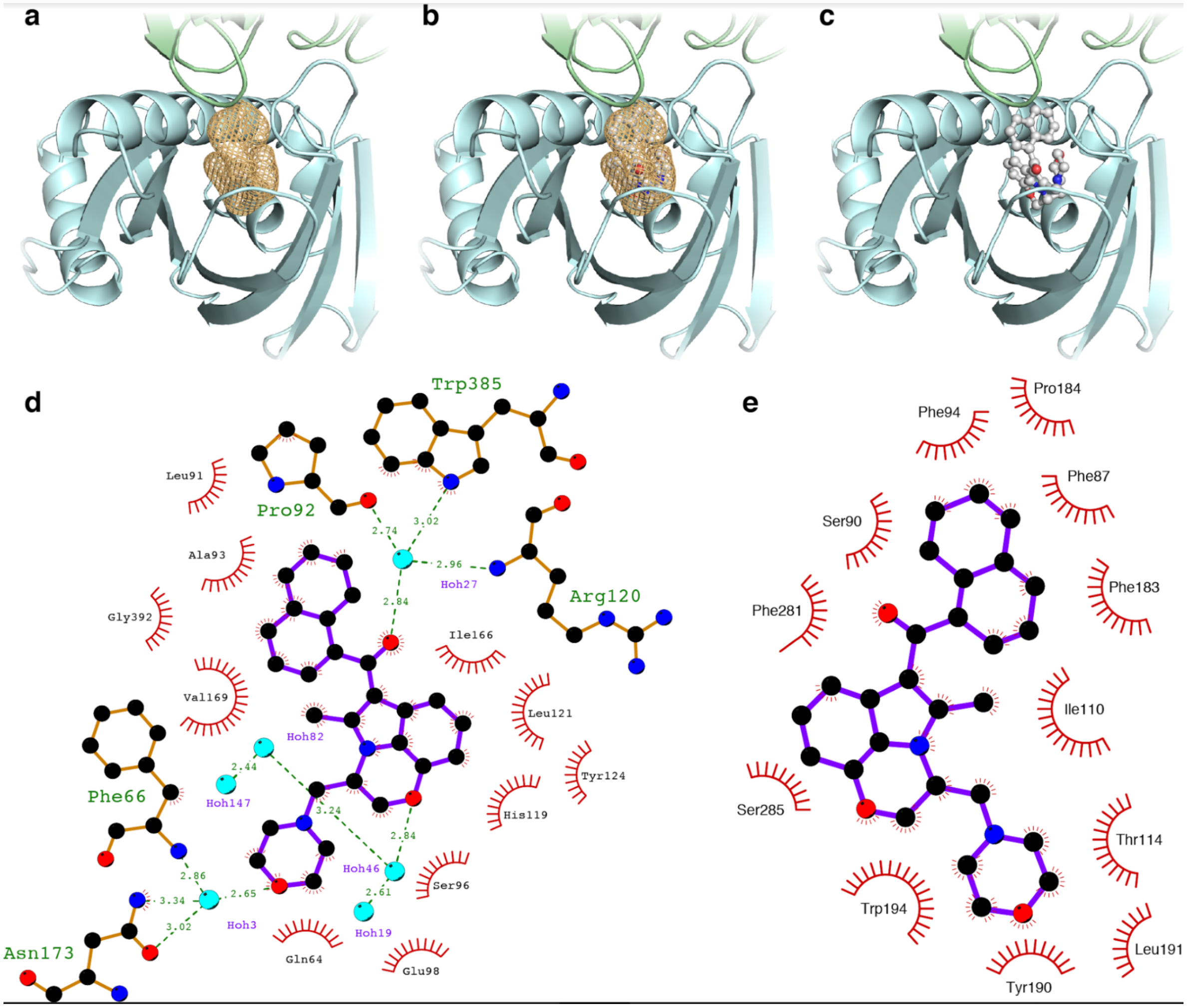

f

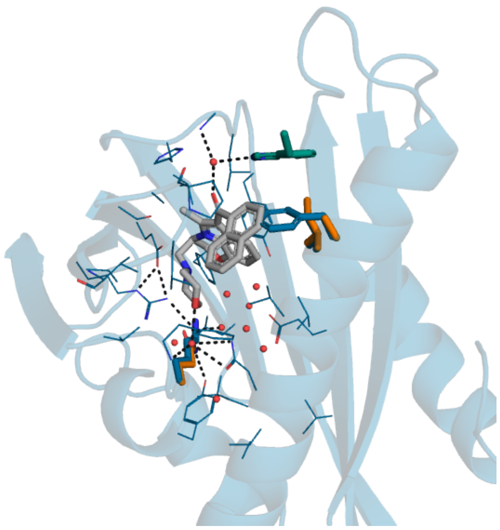

Supplemental Figure S4.

Electron density of WIN 55,212-2 in the PYL2^{WIN} ligand binding pocket**.** Several rounds of structural refinement (**a**) were carried out before modeling WIN 55,212-2 into the ligand binding pocket's unbiased electron density (**b** + **c**); a real-space correlation coefficient of 0.967 calculated between the unbiased electron density and (+)-WIN 55,212-2 indicates agreement between the model and observed electron density. Ligplot illustrating hydrophobic and water-mediated contacts between WIN 55212-2 and the engineered PYL2^{WIN} receptor (**d**) in comparison to the hydrophobic contacts that stabile WIN 55212-2 binding to the human cannabinoid receptor CB2 (**e**) (PDB 6PT0). The binding poses (conformations) of WIN 55212-2 also differ substantially between the two structures (see main Figure 2e). (**f**) Water-mediated hydrogen bonds connecting PYL2^{WIN} and ligand are realized through K59Q, which rearranges the extensive hydrogen bond network at the base of the pocket.

Top

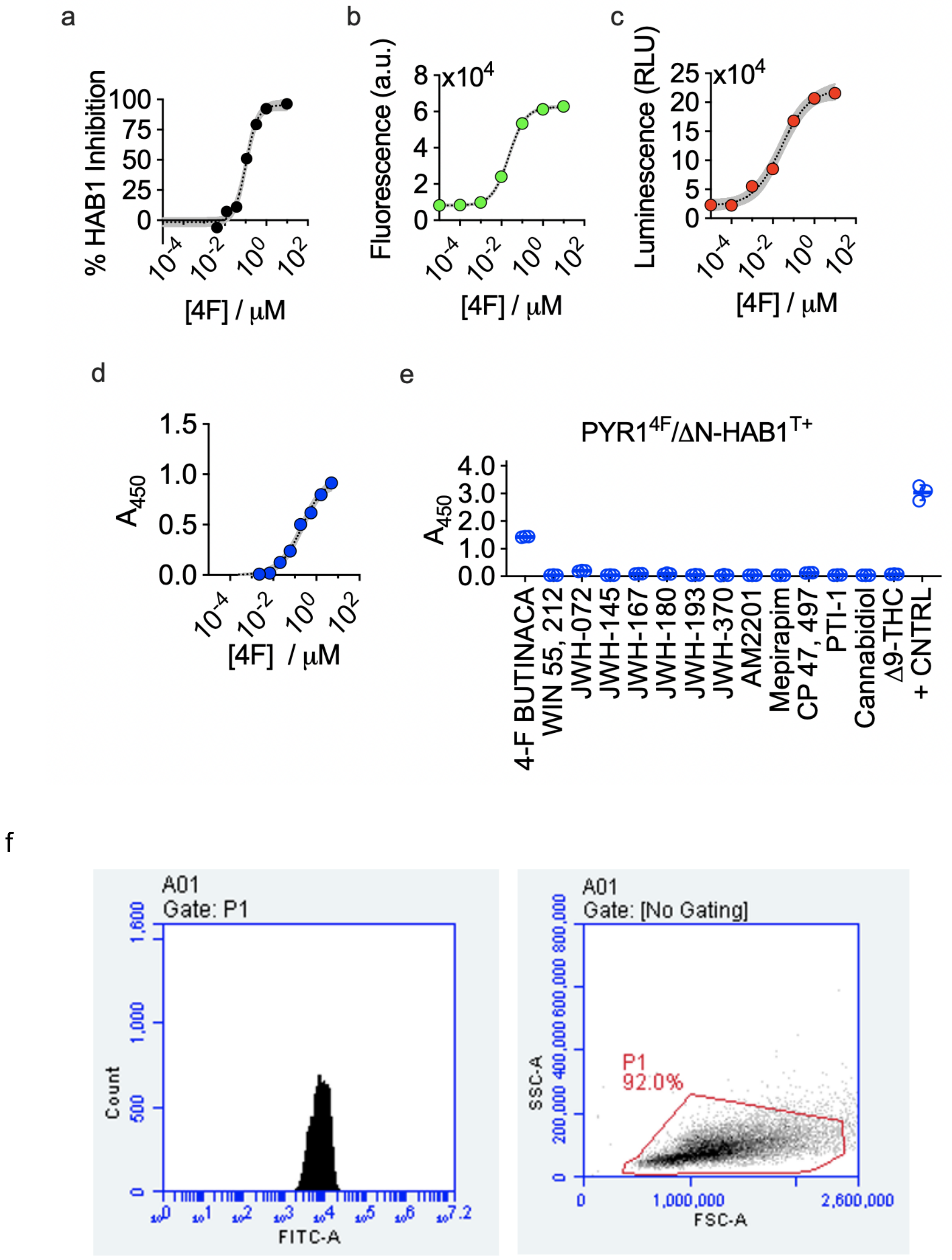
a
% HAB1 Inhibition
100
75
50
25
0
10-4
10-2
100
102
[4F] / μM
b
Fluorescence (a.u.)
8 x10^4
6
4
2
0
[4F] / μM
c
Luminescence (RLU)
25 x10^4
20
15
10
5
0
[4F] / μM
d
A450
1.5
1.0
0.5
0.0
[4F] / μM
e
PYR1^4F/ΔN-HAB1^T+
A450
4.0
3.0
2.0
1.0
0.0
4-F BUTINACA
WIN 55, 212
JWH-072
JWH-145
JWH-167
JWH-180
JWH-193
JWH-370
AM2201
Mepirapim
CP 47, 497
PTI-1
Cannabidiol
Δ9-THC
+ CNTRL
f
A01
Gate: P1
Count
FITC-A
A01
Gate: [No Gating]
P1
92.0%
SSC-A
FSC-A

Middle

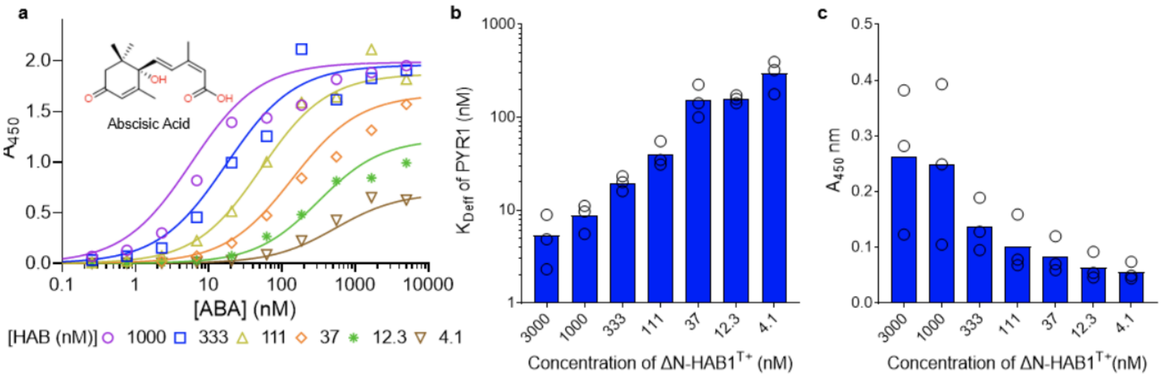

Bottom

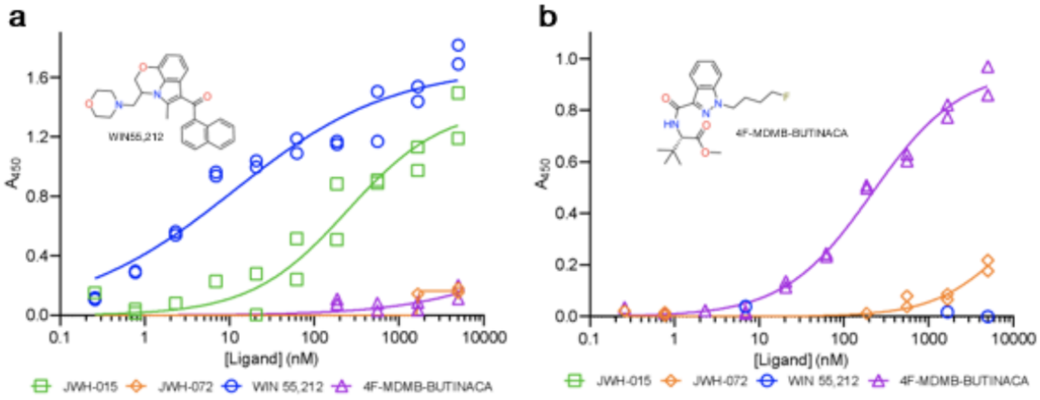

Supplemental Figure S5.

(top) PYR1-based sensors are portable to diverse CID-based output systems demonstrated with PYR1^{4F}. (**a**) *Phosphatase inhibition*. Ligand-dependent inhibition of ΔN-HAB1 phosphatase activity by recombinant receptors using a fluorogenic substrate. Inhibition expressed relative to mock controls (n = 3). (**b**) *Gene activation.* Ligand-induced gene activation in *S. cerevisiae* using an engineered $Z4_{DBD}$-PYR1/$VP64_{AD}$-ΔN-HAB1 genetic circuit. Whole-cell fluorescence generated from an integrated $Z4_4$-$CYC1_{core}$-GFP-CYC1t reporter is shown (12 h post ligand addition; n = 3). (**c**) *Split luciferase complementation*. Addition of 4F results in luminescence from Luc^N-PYR1^{4F}/ Luc^C-ΔN-HAB1 (n = 3). (**d**) *PYR1 ELISA-like immunoassays*. Immobilized receptors recruit biotinylated ΔN-HAB1^{T+} in response to ligand and colorimetric signal is generated by a secondary streptavidin HRP conjugate (n=3). Data points represent the mean and the 95% confidence interval (c.i.) is shown on fits in **a, b, c,** & **d** as grey shading. (**e**) Receptor cross-reactivity evaluation in PYR1 ELISAs. The cannabinoids shown were assayed for signal generation at 2 µM. "+ CNTRL" is PYR1^M tested with 2 µM ABA. (n=3). (**f**) Example of the gating strategy used to analyze flow cytometry data shown in part b. A minimum of 10,000 events were analyzed in each sample.

(middle) Reconstitution and optimization of the PYR1/HAB1 CID system in a plate-based ELISA. (**a**) Symbols represent empirical titration curves of ABA with increasing concentrations of ΔN-HAB1^{T+} in the binding assay with immobilized PYR1. Lines indicate global best fit based upon the empirical data. Concentrations of HAB used are indicated as follows: purple circles are 1000 nM, blue squares are 333 nM, gold up-pointing triangles are 111 nM, orange diamonds are 37 nM, green starbursts are 12.3 nM, and brown down-triangles are 4.1 nM. The structure of ABA is shown. (**b**) Effective K_D values for the PYR1 / ABA / ΔN-HAB1^{T+} system in a plate-based ELISA assay, using the data from Panel a. Data points indicate three independent biological replicates performed on separate days. Note the log scale. (**c**) Background signal generated

from interaction of PYR1 and ΔN-HAB1^{T+} in the absence of ABA, shown from the same biological replicates used for panels a. and b.. Note the linear scale. Experiments were repeated on three separate days.

(bottom) Reconstitution of the PYR/HAB based CID system in a plate-based ELISA. Experiments were repeated on two separate days and data points indicate values for both technical replicates within one of the independent days (n = 4 total). (**a**) Titration curve of a select subset of cannabinoids with immobilized PYR$^{WIN\text{-}M}$ and 1 μM ΔN-HAB1^{T+} . Ligands are indicated as follows: blue circles are WIN55,212, green squares are JWH-015, orange diamonds are JWH-072, and purple triangles are 4F-MDMB-BUTINACA. Data shown with "No ligand" control subtracted. Structure of the target ligand, WIN55,212 is shown.(**b**) Titration curve of a select subset of cannabinoids with immobilized PYR$^{4F\text{-}M}$ and 1 μM ΔN-HAB1^{T+}. Ligands are indicated as follows: blue circles are WIN55,212, green squares are JWH-015, orange diamonds are JWH-072, and purple triangles are 4F-MDMB-BUTINACA. Data shown with "No ligand" control subtracted. Structure of the target ligand, 4F-MDMB-BUTINACA is shown.

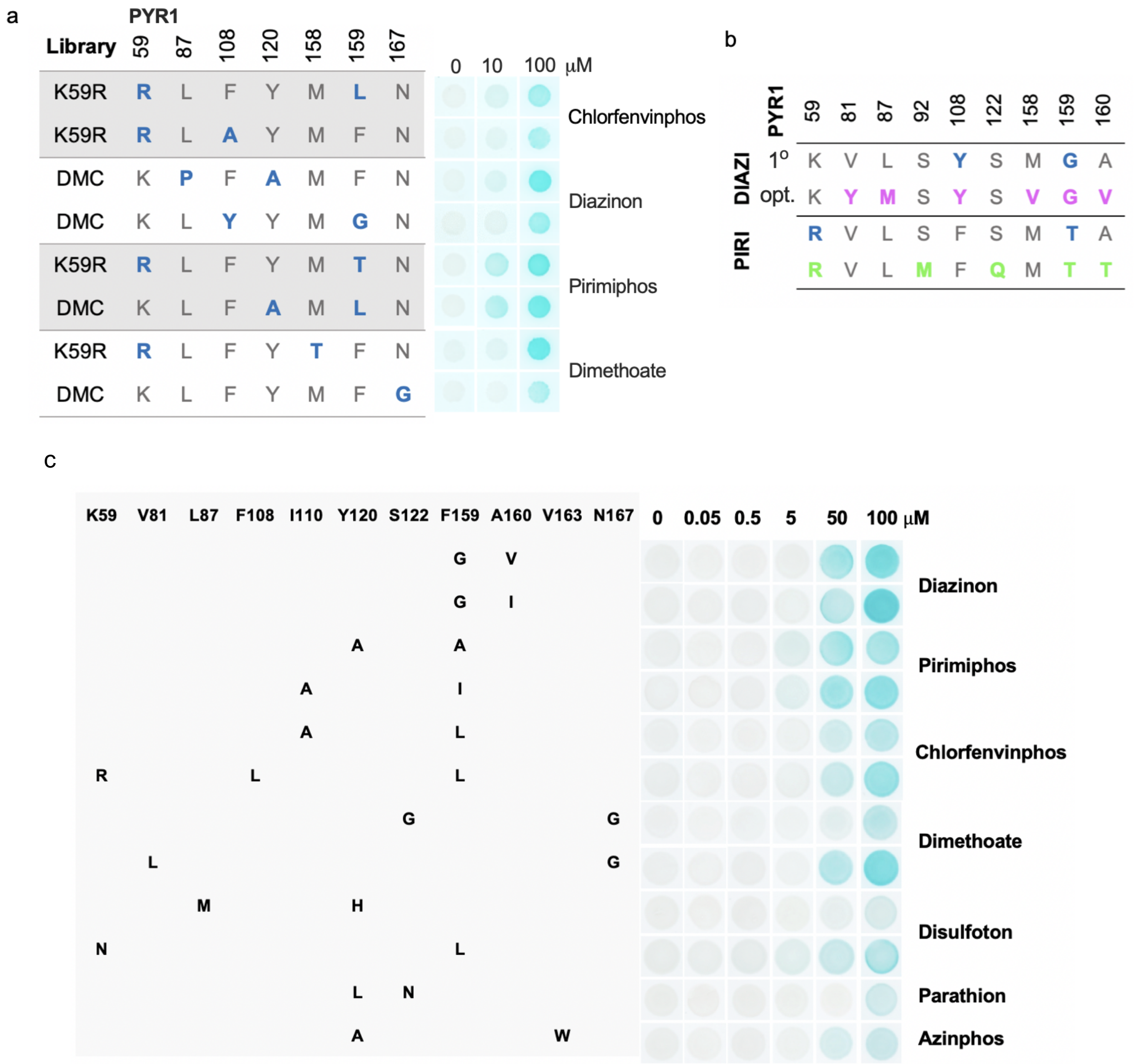

Supplementary Figure S6. Initial hits and optimization of organophosphate PYR1-based sensors.

(a) Mutations and Y2H data of the initial organophosphate hits. The library used for screening is indicated on the left.

(b) Binding pocket mutations of optimized diazinon and pirimiphos receptors. Binding pocket mutations in the primary hit are also shown.

(c) The DSM library was screened at 100 μM concentrations of the organophosphate panel. This led to the identification of PYR1 mutants for 7 different organophosphates, the mutations present in each mutant are shown at left. Chemical dose responses (Y2H assay) were conducted for two different mutant receptors per chemical, except parathion and azinphos for which only a single receptor was identified. The mutations present in all DSM mutant receptors identified hits are shown in Supplemental Data File 2.

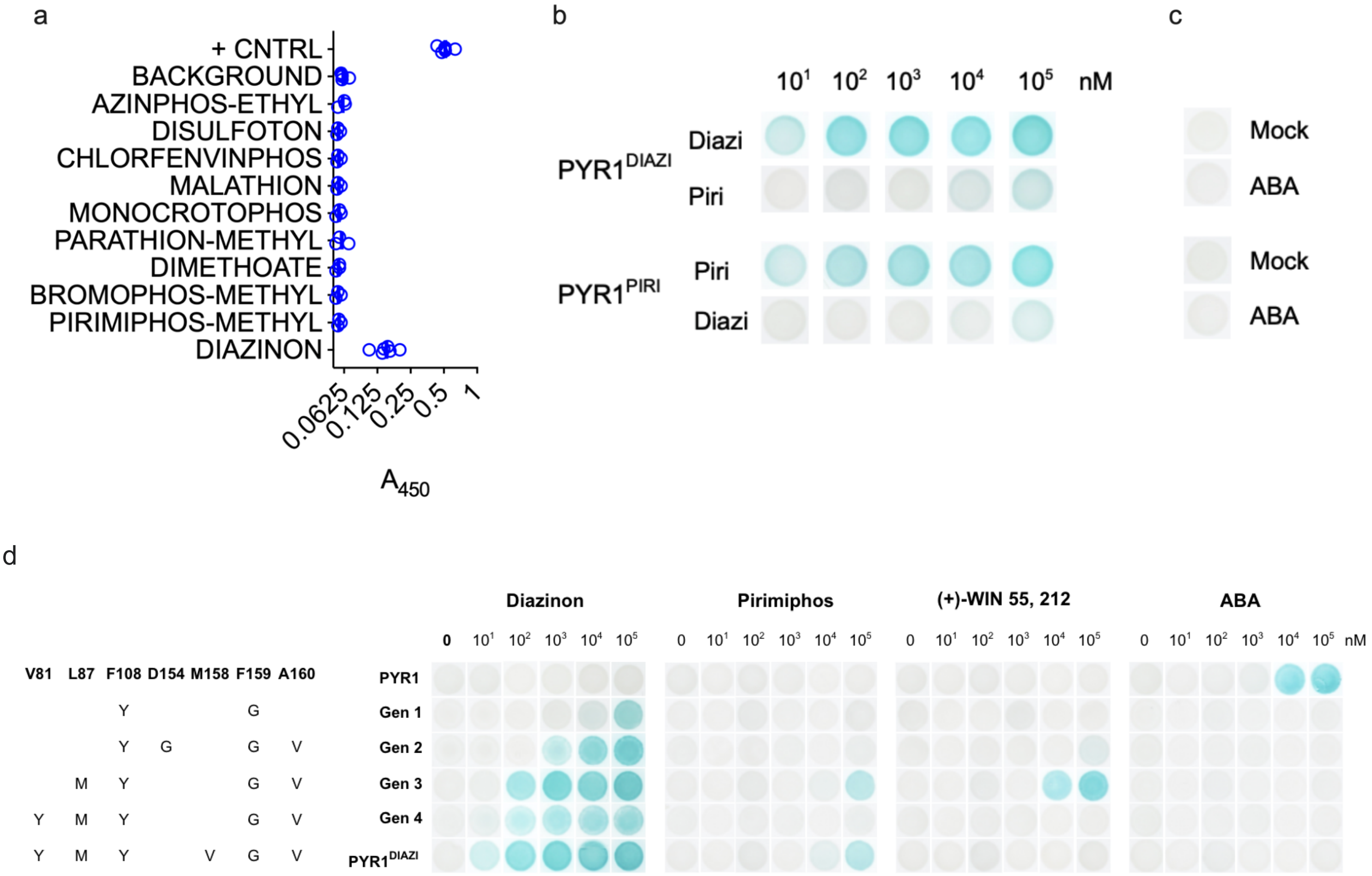

Supplementary Figure S7.

(a) Receptor cross-reactivity evaluation in PYR1 ELISAs. The organophosphates shown were assayed for signal generation at 20 µM. "+ CNTRL" is $PYR1^{M}$ tested with 20 µM ABA. "BACKGROUND" is the A450 signal for the $PYR1^{DIAZI}$ sensor in the absence of ligand (n=3 for all samples except for n=5 (BACKGROUND); n=6 (DIAZINON; + CNTRL)).

(b) Yeast-two hybrid-based assays of $PYR1^{DIAZI}$ and $PYR1^{PIRI}$ cross-reactivity to on- and off-target ligands (tested at the concentrations shown).

(c) Yeast-two hybrid-based assays of basal and ABA cross-reactivity of $PYR1^{DIAZI}$ and $PYR1^{PIRI}$ sensors. ABA tested at 100 µM. Neither $PYR1^{DIAZI}$ nor $PYR1^{PIRI}$ are activated by ABA.

(d) Specificity test of $PYR1^{DIAZI}$ optimization clones. Gain of function PYR1 variants resulted from each generation of affinity optimization (Gen1 –$PYR1^{DIAZI}$) to diazinon were tested for cross-reactivity with pirimiphos-methyl, (+)-WIN 55, 212 and ABA. Results suggest that sensitive and selective sensors can be obtained (i.e, Gen 4 and $PYR1^{DIAZI}$) despite induced promiscuity in some intermediate variants.

TABLES

Table S1. Data collection and refinement statistics

	PYL2:WIN: ΔN-HAB1^{T+}
**Data collection**	
Space group	P 21 21 21
Cell dimensions	
a, *b*, *c* (Å)	61.33, 69.23, 142.82
α, *β*, *γ* (°)	90, 90, 90
Resolution (Å)	50-1.902 (1.97-1.902)
Total Reflections	351,970 (34628)
Unique Reflections	48,429 (4,740)
R_{merge}	0.060 (1.270)
R_{meas}	0.065 (1.366)
R_{pim}	0.024 (0.499)
CC1/2	0.999 (0.756)
*CC**	1.000 (0.928)
$I / \sigma I$	16.7 (1.7)
Completeness (%)	99.6 (98.8)
Redundancy	7.3 (57.3)
**Refinement**	
Resolution (Å)	38.61-1.902 (1.97-1.902)
No. reflections	48,408 (4,740)
R_{cryst}	0.185 (0.326)
R_{free}	0.211 (0.333)
No. atoms	3967
Protein	3685
Ligand/ion	63
Water	219
B-factors	
Protein	53.3
Ligand/ion	46.1
Water	50.2
R.m.s. deviations	
Bond lengths (Å)	0.007
Bond angles (°)	0.770
Ramachandran Stats	
Favored	98.1%
Allowed	1.9%

Values in parentheses are for highest-resolution shell.
A single crystal was used for each structure determination.

References

Adams, Paul D., Pavel V. Afonine, Gábor Bunkóczi, Vincent B. Chen, Ian W. Davis, Nathaniel Echols, Jeffrey J. Headd, et al. 2010. “PHENIX: A Comprehensive Python-Based System for Macromolecular Structure Solution.” *Acta Crystallographica. Section D, Biological Crystallography* 66 (Pt 2): 213–21.

Davis, Ian W., Andrew Leaver-Fay, Vincent B. Chen, Jeremy N. Block, Gary J. Kapral, Xueyi Wang, Laura W. Murray, et al. 2007. “MolProbity: All-Atom Contacts and Structure Validation for Proteins and Nucleic Acids.” *Nucleic Acids Research* 35 (Web Server issue): W375–83.

Dupeux, Florine, Julia Santiago, Katja Betz, Jamie Twycross, Sang-Youl Park, Lesia Rodriguez, Miguel Gonzalez-Guzman, et al. 2011. “A Thermodynamic Switch Modulates Abscisic Acid Receptor Sensitivity.” *The EMBO Journal* 30 (20): 4171–84.

Elzinga, Dezi, Erin Sternburg, Davide Sabbadin, Michael Bartsch, Sang-Youl Park, Aditya Vaidya, Assaf Mosquna, et al. 2019. “Defining and Exploiting Hypersensitivity Hotspots to Facilitate Abscisic Acid Receptor Agonist Optimization.” *ACS Chemical Biology*, January. https://doi.org/10.1021/acschembio.8b00955.

Emsley, Paul, and Kevin Cowtan. 2004. “Coot: Model-Building Tools for Molecular Graphics.” *Acta Crystallographica. Section D, Biological Crystallography* 60 (Pt 12 Pt 1): 2126–32.

Kabsch, Wolfgang. 2010. “XDS.” *Acta Crystallographica. Section D, Biological Crystallography* 66 (Pt 2): 125–32.

Kowalsky, Caitlin A., and Timothy A. Whitehead. 2016. “Determination of Binding Affinity upon Mutation for Type I Dockerin-Cohesin Complexes from Clostridium Thermocellum and Clostridium Cellulolyticum Using Deep Sequencing.” *Proteins* 84 (12): 1914–28.

Lin, Hsin-Ying, Sey-En Lin, Su-Fang Chien, and Ming-Kai Chern. 2011. “Electroporation for Three Commonly Used Yeast Strains for Two-Hybrid Screening Experiments.” *Analytical Biochemistry* 416 (1): 117–19.

Medina-Cucurella, Angélica V., Paul J. Steiner, Matthew S. Faber, Jesús Beltrán, Alexandra N. Borelli, Monica B. Kirby, Sean R. Cutler, and Timothy A. Whitehead. 2019. “User-Defined Single Pot Mutagenesis Using Unamplified Oligo Pools.” *Protein Engineering, Design & Selection: PEDS* 32 (1): 41–45.

Melcher, Karsten, Ley-Moy Ng, X. Edward Zhou, Fen-Fen Soon, Yong Xu, Kelly M. Suino-Powell, Sang-Youl Park, et al. 2009. “A Gate-Latch-Lock Mechanism for Hormone Signalling by Abscisic Acid Receptors.” *Nature* 462 (7273): 602–8.

Mosquna, Assaf, Francis C. Peterson, Sang-Youl Park, Jorge Lozano-Juste, Brian F. Volkman, and Sean R. Cutler. 2011. “Potent and Selective Activation of Abscisic Acid Receptors in Vivo by Mutational Stabilization of Their Agonist-Bound Conformation.” *Proceedings of the National Academy of Sciences of the United States of America* 108 (51): 20838–43.

Müller, Kristian M., Sabine C. Stebel, Susanne Knall, Gregor Zipf, Hubert S. Bernauer, and Katja M. Arndt. 2005. “Nucleotide Exchange and Excision Technology (NExT) DNA Shuffling: A Robust Method for DNA Fragmentation and Directed Evolution.” *Nucleic Acids Research* 33 (13): e117.

Mumberg, D., R. Müller, and M. Funk. 1995. “Yeast Vectors for the Controlled Expression of Heterologous Proteins in Different Genetic Backgrounds.” *Gene* 156 (1): 119–22.

Park, Sang-Youl, Francis C. Peterson, Assaf Mosquna, Jin Yao, Brian F. Volkman, and Sean R. Cutler. 2015. “Agrochemical Control of Plant Water Use Using Engineered Abscisic Acid Receptors.” *Nature* 520 (7548): 545–48.

Schüttelkopf, Alexander W., and Daan M. F. van Aalten. 2004. “PRODRG: A Tool for High-Throughput Crystallography of Protein–ligand Complexes.” *Acta Crystallographica Section D Biological Crystallography*. https://doi.org/10.1107/s0907444904011679.

Steiner, Paul J., Zachary T. Baumer, and Timothy A. Whitehead. 2020. “A Method for User-Defined Mutagenesis by Integrating Oligo Pool Synthesis Technology with Nicking Mutagenesis.” *Bio-Protocol* 10 (15): e3697.

Steiner, Paul J., Matthew A. Bedewitz, Angélica V. Medina-Cucurella, Sean R. Cutler, and Timothy A. Whitehead. 2020. “A Yeast Surface Display Platform for Plant Hormone Receptors: Toward Directed Evolution of New Biosensors.” *AIChE Journal. American Institute of Chemical Engineers* 66 (3). https://doi.org/10.1002/aic.16767.

Vaidya, Aditya S., Jonathan D. M. Helander, Francis C. Peterson, Dezi Elzinga, Wim Dejonghe, Amita Kaundal, Sang-Youl Park, et al. 2019. “Dynamic Control of Plant Water Use Using Designed ABA Receptor Agonists.” *Science* 366 (6464). https://doi.org/10.1126/science.aaw8848.

Wrenbeck, Emily E., Justin R. Klesmith, James A. Stapleton, Adebola Adeniran, Keith E. J. Tyo, and Timothy A. Whitehead. 2016. “Plasmid-Based One-Pot Saturation Mutagenesis.” *Nature Methods* 13 (11): 928–30.
