# Supplementary material for: Rapid biosensor development using plant hormone receptors as reprogrammable scaffolds
Source: Nat Biotechnol. 2022 Jun 20;40(12):1855–61. doi: 10.1038/s41587-022-01364-5 (PMC9750858; doi:10.1038/s41587-022-01364-5)
Supplement: Supplementary file 2 — Reporting Summary [file 41587_2022_1364_MOESM2_ESM.pdf]

nature portfolio

Corresponding author(s): Whitehead, Cutler, Wheeldon

Last updated by author(s): Apr 26, 2022

Reporting Summary

Nature Portfolio wishes to improve the reproducibility of the work that we publish. This form provides structure for consistency and transparency in reporting. For further information on Nature Portfolio policies, see our Editorial Policies and the Editorial Policy Checklist.

Statistics

For all statistical analyses, confirm that the following items are present in the figure legend, table legend, main text, or Methods section.

n/a	Confirmed	
☐	☒	The exact sample size (n) for each experimental group/condition, given as a discrete number and unit of measurement
☐	☒	A statement on whether measurements were taken from distinct samples or whether the same sample was measured repeatedly
☐	☒	The statistical test(s) used AND whether they are one- or two-sided<br>*Only common tests should be described solely by name; describe more complex techniques in the Methods section.*
☒	☐	A description of all covariates tested
☒	☐	A description of any assumptions or corrections, such as tests of normality and adjustment for multiple comparisons
☐	☒	A full description of the statistical parameters including central tendency (e.g. means) or other basic estimates (e.g. regression coefficient) AND variation (e.g. standard deviation) or associated estimates of uncertainty (e.g. confidence intervals)
☒	☐	For null hypothesis testing, the test statistic (e.g. F, t, r) with confidence intervals, effect sizes, degrees of freedom and P value noted<br>*Give P values as exact values whenever suitable.*
☒	☐	For Bayesian analysis, information on the choice of priors and Markov chain Monte Carlo settings
☒	☐	For hierarchical and complex designs, identification of the appropriate level for tests and full reporting of outcomes
☒	☐	Estimates of effect sizes (e.g. Cohen's d, Pearson's r), indicating how they were calculated

Our web collection on statistics for biologists contains articles on many of the points above.

Software and code

Policy information about availability of computer code

Data collection	BD Accuri C6+ analysis software or FlowJo software were used to collect flow cytometry data. No custom code was used to collect data.
Data analysis	Statistical analysis for all graphical data presented was performed on Graphpad Prism version 9.0. Custom scripts were used for deep sequencing data analysis; these scripts are available on Zenodo under DOI: 10.5281/zenodo.6493839

For manuscripts utilizing custom algorithms or software that are central to the research but not yet described in published literature, software must be made available to editors and reviewers. We strongly encourage code deposition in a community repository (e.g. GitHub). See the Nature Portfolio guidelines for submitting code & software for further information.

Data

Policy information about availability of data

All manuscripts must include a data availability statement. This statement should provide the following information, where applicable:

- Accession codes, unique identifiers, or web links for publicly available datasets
- A description of any restrictions on data availability
- For clinical datasets or third party data, please ensure that the statement adheres to our policy

Accession code for the WIN-PYL2WIN-HAB1T+ structure is listed as 7MWN. Libraries will be made available through AddGene pending University approval.

March 2021

Field-specific reporting

Please select the one below that is the best fit for your research. If you are not sure, read the appropriate sections before making your selection.

☒ Life sciences ☐ Behavioural & social sciences ☐ Ecological, evolutionary & environmental sciences

For a reference copy of the document with all sections, see nature.com/documents/nr-reporting-summary-flat.pdf

Life sciences study design

All studies must disclose on these points even when the disclosure is negative.

Sample size	No sample size calculations were performed. The sample size (n) of each experiment is provided in the corresponding figure captions or methods section in the main manuscript and supplementary information files. Sample sizes were chosen to support meaningful conclusions.
Data exclusions	No data were excluded
Replication	All data are presented in biological duplicate or triplicate as noted in the figure legends.
Randomization	The work does not involve participant groups; therefore, randomization was not relevant the study.
Blinding	The work does not involve participant groups, therefore, blinding was not relevant to the study.

Reporting for specific materials, systems and methods

We require information from authors about some types of materials, experimental systems and methods used in many studies. Here, indicate whether each material, system or method listed is relevant to your study. If you are not sure if a list item applies to your research, read the appropriate section before selecting a response.

Materials & experimental systems

n/a	Involved in the study	
☐	☒	Antibodies
☒	☐	Eukaryotic cell lines
☒	☐	Palaeontology and archaeology
☒	☐	Animals and other organisms
☒	☐	Human research participants
☒	☐	Clinical data
☒	☐	Dual use research of concern

Methods

n/a	Involved in the study	
☒	☐	ChIP-seq
☐	☒	Flow cytometry
☒	☐	MRI-based neuroimaging

Antibodies

Antibodies used	anti-cmyc FITC was used for yeast surface display measurements in Figure S4. Details of this reagent are provided in the materials section of the supporting information.
Validation	anti-cmyc FITC (part no. 130-116-653 used in these studies) was validated as described by the commercial vendor here -- https://www.miltenyibiotec.com/US-en/products/c-myc-antibody-anti-human-mouse-rat-sh1-26e7-1-3.html#fitc:30-tests-in-60-ul

Flow Cytometry

Plots

Confirm that:

☒ The axis labels state the marker and fluorochrome used (e.g. CD4-FITC).

☒ The axis scales are clearly visible. Include numbers along axes only for bottom left plot of group (a 'group' is an analysis of identical markers).

☒ All plots are contour plots with outliers or pseudocolor plots.

☒ A numerical value for number of cells or percentage (with statistics) is provided.

Methodology

Sample preparation	The cells were washed with 1 mL PBS buffer twice and resuspended in 1 mL DI water for flow cytometry analysis. For analysis,

Sample preparation	50 uL of resuspended cells were transferred to a 96-well plate with flat bottom, adding DI water up to a final volume of 200 uL.
Instrument	Samples were analyzed using either a BD Accuri C6+ flow cytometry or Sony Biotech SH800.
Software	Samples were analyzed using either BD Accuri C6+ analysis software or FlowJo software.
Cell population abundance	At least 10,000 cells were analyzed in each experiment, gating data is shown in the supporting information.
Gating strategy	The standard gating for yeast measurements has been adequately described in previous publications, e.g., Medina-Cucurella A and Whitehead TA (2018) "Characterizing Protein-Protein Interactions Using Deep Sequencing Coupled to Yeast Surface Display", Methods in Molecular Biology in Protein Complex Assembly, 101-121.; the full gating strategy is shown in the SI.

☒ Tick this box to confirm that a figure exemplifying the gating strategy is provided in the Supplementary Information.
